# Health assessment of future PM_2.5_ exposures from indoor, outdoor, and secondhand tobacco smoke concentrations under alternative policy pathways in Ulaanbaatar, Mongolia

**DOI:** 10.1371/journal.pone.0186834

**Published:** 2017-10-31

**Authors:** L. Drew Hill, Rufus Edwards, Jay R. Turner, Yuma D. Argo, Purevdorj B. Olkhanud, Munkhtuul Odsuren, Sarath Guttikunda, Chimedsuren Ochir, Kirk R. Smith

**Affiliations:** 1 Division of Environmental Health Sciences, University of California, Berkeley, CA, United States of America; 2 Department of Epidemiology, University of California Irvine, Irvine, CA, United States of America; 3 Department of Energy, Environmental, and Chemical Engineering, Washington University, St. Louis, MO, United States of America; 4 School of Public Health, Mongolian National University of Medical Sciences, Ulaanbaatar, Mongolia; 5 Independent Consultant, San Francisco, CA, United States of America and Ulaanbaatar, Mongolia; 6 Sickle Cell Branch, National Heart, Lung, and Blood Institute, National Institutes of Health, Bethesda, MD, United States of America; 7 Division of Atmospheric Sciences, Desert Research Institute, Reno, NV, United States of America; Utah State University, UNITED STATES

## Abstract

**Introduction:**

Winter air pollution in Ulaanbaatar, Mongolia is among the worst in the world. The health impacts of policy decisions affecting air pollution exposures in Ulaanbaatar were modeled and evaluated under business as usual and two more-strict alternative emissions pathways through 2024. Previous studies have relied on either outdoor or indoor concentrations to assesses the health risks of air pollution, but the burden is really a function of total exposure. This study combined projections of indoor and outdoor concentrations of PM_2.5_ with population time-activity estimates to develop trajectories of total age-specific PM_2.5_ exposure for the Ulaanbaatar population. Indoor PM_2.5_ contributions from secondhand tobacco smoke (SHS) were estimated in order to fill out total exposures, and changes in population and background disease were modeled. The health impacts were derived using integrated exposure-response curves from the Global Burden of Disease Study.

**Results:**

Annual average population-weighted PM_2.5_ exposures at baseline (2014) were estimated at 59 μg/m^3^. These were dominated by exposures occurring indoors, influenced considerably by infiltrated outdoor pollution. Under current control policies, exposures increased slightly to 60 μg/m^3^ by 2024; under moderate emissions reductions and under a switch to clean technologies, exposures were reduced from baseline levels by 45% and 80%, respectively. The moderate improvement pathway decreased per capita annual disability-adjusted life year (DALY) and death burdens by approximately 40%. A switch to clean fuels decreased per capita annual DALY and death burdens by about 85% by 2024 with the relative SHS contribution increasing substantially.

**Conclusion:**

This study demonstrates a way to combine estimated changes in total exposure, background disease and population levels, and exposure-response functions to project the health impacts of alternative policy pathways. The resulting burden analysis highlights the need for aggressive action, including the elimination of residential coal burning and the reduction of current smoking rates.

## Introduction

Mongolia’s capital city, Ulaanbaatar (UB), is home to nearly half of the nation’s three million residents. Driven initially by political-economic changes incurred after the fall of the Soviet Union and hastened by periodic bouts of harsh weather and famine [1], called dzud, UB is now experiencing rapid rural-to-urban migration and an increasing demand for coal. Between 1990 and 2014, the population of UB grew from 570,000 people (~27% of the Mongolian population) to nearly 1.4 million (~46% of the population) [2]. These factors, together, lead to winter outdoor air pollution concentrations in UB that are among the worst in the world. About half of UB residents currently live in houses or gers—traditional yurt-like dwellings—heated by simple, coal-fired stoves. Recent measurements of fine particulate matter (PM_2.5_) have shown citywide wintertime average concentrations as high as 250 μg/m^3^ and annual average outdoor concentrations that are over seven times higher than the World Health Organization (WHO) health-based guidelines established to minimize morbidity and mortality risk and three times higher than Mongolian national standards [3,4]. Daily averages are often even higher; between January-February 2016, the United States Embassy in UB reported 20% of 24-hour average values greater than 400 μg/m^3^ [5].

The high air pollution levels in UB arise from a combination of high anthropogenic emissions, geography, and meteorology [3,4]. Geographically, the city lies in a valley surrounded on three sides by mountain slopes onto which the peri-urban areas of the city have spread. Although these districts are referred to as the “ger areas,” approximately 60% of the dwellings in these districts are one and two-story houses constructed by local residents. In winter months, UB is strongly affected by the mid-continental Siberian anticyclone, a semi-permanent high-pressure system that is characterized by stagnant air masses centered over northern Mongolia. The anticyclone forms because of intense cooling of the surface layers of air over the continent during this season, leading to a well-developed temperature inversion in the lower atmosphere. These two factors by themselves would not lead to severe air pollution without high pollutant emissions largely caused by the burning of raw coal for winter heating. Recent analyses estimate that about two thirds of the annual average PM_2.5_ concentration in UB is from combustion sources with nominally equal contributions from coal-fired power plants and residential heating [6]. However, this split seems unlikely given the power plants have tall stacks which release emissions above the very shallow wintertime inversion layers. In winter, up to 70% of PM_2.5_ emissions in the ger areas are attributable to residential heating [7]. Although UB’s vehicle fleet is rapidly increasing [3], the contribution to ambient PM_2.5_ concentrations is estimated to be only in the range of 5–10% [6] due to the large impact of coal emissions. Ultimately, once fine particulate matter is emitted into the valley, its dispersion is constrained by a very low atmospheric mixing height [8].

The Mongolian government has undertaken efforts in this decade to reduce air pollution, including the subsidization of cleaner-burning coal stoves, elimination of many institutional heat only boilers (HOB), and the promotion of energy efficiency [9]. However, expansion of the peri-urban areas combined with the use of high-emission residential heating stoves continues to produce high PM_2.5_ concentrations in UB, especially in winter. Current residents are facing exposures never before experienced by Mongolians, and urgent measures are needed to reduce consequent health impacts. A 2013 analysis [3] of outdoor concentrations in UB conservatively estimated that PM_2.5_ was responsible for 29% of cardiopulmonary deaths, 40% of lung cancer deaths, and nearly 10% of all-cause mortality. The World Bank [10] places the greatest health insults in ger areas, where UB’s poorest and most vulnerable populations reside, and project steady increases in medical and economic impacts into the foreseeable future without significant policy changes. Leaders agree with the need to eventually reduce emissions across all energy sectors, but the benefits of doing so quickly rather than more slowly, a choice with substantial differences in costs and strategy, have remained unclear. To assist Mongolia’s policymakers at this critical juncture, we modeled future air pollution exposures and related health burdens in UB through 2024 under business as usual (BAU) and two alternative energy policy pathways: moderate emissions reductions across power, heating, and vehicle sectors (Pathway 1); and a major shift in these same sectors to clean fuels and technologies (Pathway 2). These pathways were based on technologies that have been successfully adopted by other countries, and represent progressively more aggressive adoption of clean fuels in order to inform on what measures may result in reduced health impacts across the population.

Our estimates improve on previous local and regional population-wide burden assessments [3,10–12] by focusing on personal exposures and incorporating local measurements of indoor concentrations and stove emissions. Our analysis employs an ambient air quality model with high spatial and temporal resolution. In contrast, hybrid satellite methods, which have been used in recent regional-scale evaluations [11,12], have coarser spatial resolution and have been found particularly unreliable in UB because of poor resolution for winter nighttime [13,14]. The use of spatiotemporally-resolved models of ambient air pollution based on local emissions allows current policies for urban development in UB to be evaluated in relation to the health impacts of alternative policy pathways. The methods developed for this analysis are among the first to incorporate the exposure-response functions of the 2010 Comparative Risk Assessments of the Global Burden of Disease Study (GBD) in a *forward-looking* analysis that focuses not only or separately on outdoor or indoor pollution, but on *total exposure* of the population.

## Methods

**Fig 1:**
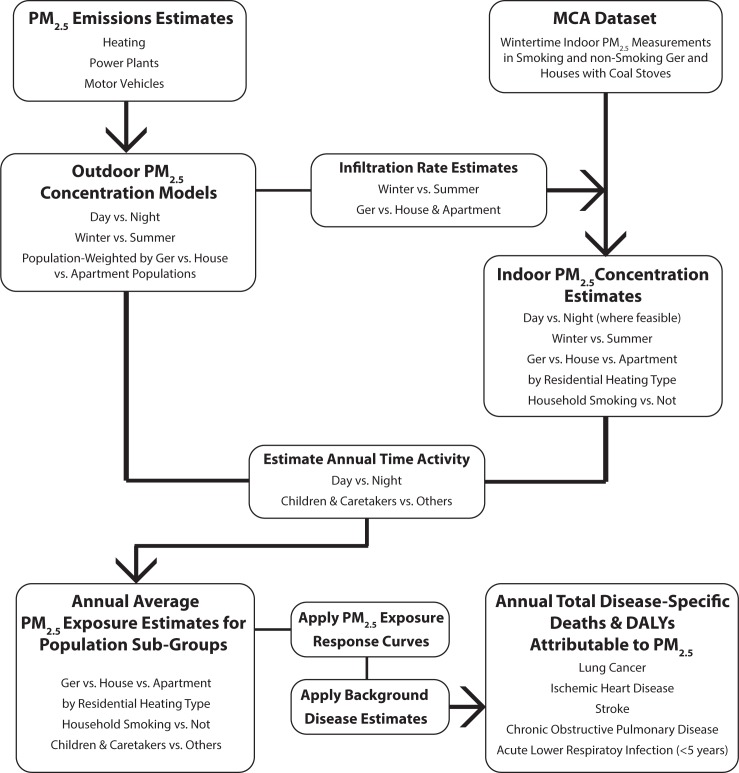
High-level flow chart of the general exposure and disease analysis approach. Annual average exposures were estimated for population sub-groups in 2014 and in 2024 under BAU and two alternative policy pathways from outdoor and indoor concentration models and time activity estimates using the approach summarized above. These exposures were applied to disease-specific exposure response curves to produce estimates of population attributable fraction (PAF) which were applied to background disease rates to quantify attributable disease burden. Detailed data descriptions and methods–including how interim year (2015–2023) disease burdens were calculated–are included in the manuscript and S1 Text.

Personal PM_2.5_ exposures and related disease burdens were modeled for UB residents through 2024 under BAU and two alternative policy pathways. Indoor and outdoor concentration estimates were combined with time-activity data, census information, demographics projections, and estimated tobacco smoking rates to provide population-weighted total exposure estimates. Fig 1 shows a summary flow chart of the exposure and disease burden calculation framework. Disease-specific estimates of health burden were produced using a modified version of the Household Air Pollution Intervention Tool [15] and projections of city-wide background disease. Data handling, mathematical modeling, and figure creation for indoor concentrations, demographic projections, exposure modeling, and health burden estimation were performed in R [16] and Microsoft Excel for Mac 2011 and 2016.

### Baseline and the pathways

Variations in the emissions trajectories of heating, power, and traffic sectors were considered in relation to policy pathways from baseline that followed business as usual or one of two alternative policy approaches: moderate restrictions in addition to those in place at baseline (Pathway 1) and additional aggressive restrictions (Pathway 2). The variations examined were most detailed for household heating, which has been identified as the single largest contributor to outdoor air pollution in UB [8]. Household heating types in Ulaanbaatar include stoves fired with raw coal; stoves fired with semi-coke fuels (semi-coke stoves), which are relevant only to houses and gers; coal-fired low pressure boilers (LPB) used to heat radiator systems in houses; and heating sources that produce no indoor emissions at the point-of-use (Clean Indoor Use, or CIU heat), such as HOB or centrally-distributed steam that is produced during combined electricity and heat generation, which can be deployed in all home types. Table 1 and Table 2 show a summary of baseline and 2024 under BAU and the two alternative energy policy pathways considered.

**Table 1:** Summary of the assumptions made for emissions sources, by category ^1^.

	Household Heating	Power Plants	Vehicles
**Baseline (2014)**	20,000 LPB-heated houses; semi-coke coal in gers & houses in Bayangol district; apartments heated with CIU heat including HOB units; MCA stoves in all other gers & houses	Four CHP: CHP-2, CHP-3 (two units), and CHP-4	Nearly 100% growth over values from 2010—the most recent inventory at the time of analysis
**BAU (2024)**	All homes, except LPB and clean-heat homes, transitioned to MCA stoves	Addition of CHP-5, which meets U.S. New Source Performance Standards	2.5%/year growth from 2014 and addition of Euro III standards
**Pathway 1 (2024)**	Transition of half of non-LPB houses to clean heat; replacement of remaining MCA stoves with "Future Tech" raw coal stove; 50% of HOB decommissioned, others retrofitted with controls	Addition of CHP-5; high-efficiency retrofits of CHP-2, CHP-3, and CHP-4.	2.5%/year growth from 2014 and addition of Euro V standards
**Pathway 2 (2024)**	Transition of all ger and houses to clean heat; all HOB decommissioned	Addition of CHP-5; CHP-3,-4 retrofitted; CHP-2 replaced by renewables and/or imports	Increased mass-transit ridership; improved traffic flow; Euro VI standards; among others

1.LPB = low pressure boiler, CIU = heating method with clean indoor use, HOB = heat only boiler, MCA = improved coal stove distributed by the Mongolian government and the U.S. Millennium Challenge Account, CHP = combined heat and power plant.

**Table 2:** Assignment of heating type of all UB households in BAU and each alternative pathway.

	*Proportion of Gers*	*Proportion of Houses*	*Proportion of Apartments*
**2014 –Baseline**	** **	** **	** **
*MCA Stove* ^*1*^	92.9%	75.2%	0%
*Stove w/Semi-Coke Fuel*	7.1%	6.0%	0%
*Low Pressure Boiler*	0%	18.8%	0%
*Heat w/Clean Indoor Use*	0%	0%	100%
**2024 –BAU**	** **	** **	** **
*MCA Stove*	100%	82.9%	0%
*Low Pressure Boiler*	0%	17.1%	0%
*Heat w/Clean Indoor Use*	0%	0%	100%
**2024 –Pathway 1**	** **	** **	** **
*Future Tech Stove*	100%	41.4%	0%
*Low Pressure Boiler*	0%	17.1%	0%
*Heat w/Clean Indoor Use*	0%	41.4%	100%
**2024 –Pathway 2**	** **	** **	** **
*Clean*	100%	100%	100%

1.Improved coal stove distributed by the Mongolian government and United States Millennium Challenge Account (MCA).

Recent efforts by the Mongolian Government, the U.S. Millennium Challenge Corporation and Millennium Challenge Account (MCA) [9], and the World Bank have aimed to replace the majority of traditional raw-coal stoves with coal stoves that substantially reduce outdoor emissions, hereafter “MCA stoves.” For this reason, complete penetration of MCA stoves was assumed in gers and houses not using LPB or semi-coke coal. At the time of the study, gers and houses in the Bayangol district were not targeted by MCA stove dissemination plans due to a concurrent semi-coke coal intervention underway in the district. Homes in this district were assumed to rely on traditional stove technology but with cleaner semi-coke coal. Based on 2012 census data 7% of all ger and 6% of all houses in the city were assigned to the Bayangol district at baseline [17]. We assumed 20,000 LPB households with one LPB per home, based on information provided to us by government officials at the Clean Air Fund. This was consistent with data showing 14,186 LPB households in 2010 [18]. All apartment households were assigned CIU heating at the household, provided either by steam heat or heat only boiler (HOB) [10]. UB currently employs four combined heat and power (CHP) plants in the generation of heat and electricity—CHP-2, CHP-3 (two units), and CHP-4. Baseline assumed this setup. Motor vehicle data for UB were sparse, and so BAU and the alternative pathways were based on plausible assumptions about trends in total fleet emissions. Values were scaled from a 2010 inventory [19] of vehicle exhaust emissions from travel on major and minor roads—this excluded emissions related to brake wear, tire wear, or re-suspended dust. Baseline PM_2.5_ emissions were estimated as 1.7 times the 2010 inventory which was the growth in the number of registered vehicles between 2010 and 2013. This is likely an overestimate because of the partial offset from a vehicle travel day ban program which requires most vehicles not be used one day per week based on license plate number.

Business as Usual assumed no major changes from trends underway at the time of the study (mid-2013) by 2024. This included a transition to MCA stoves of all homes not employing clean heat or LPB at baseline. Because HOB and LPB are outdated technologies, no net increases in the number of HOBs or LPB-heated homes were assumed. BAU retained the power plant emissions in 2024 and included a new 820 MW power plant (CHP-5) to be located 15km east of the UB Central Business District, as supported by recent government plans to develop a 450 MW plant and expand it to 820 MW shortly thereafter [20,21]. CHP-5 was assumed to meet the U.S. New Source Performance Standards for electric utility power plants. Vehicle emissions assumed a growth of 1.3 times the 2014 inventory based on an emissions growth rate of 2.5%/year over the 10-year period and Euro III emissions standards–a higher growth rate seemed unreasonable given the existing transportation network infrastructure.

Pathway 1, or moderate emissions reductions, assumed all changes in BAU as well as some moderate improvements. All 20,000 LPB-heated homes from baseline remained as such under Pathway 1. Remaining non-LPB houses were assumed equally split between clean heating and an even cleaner hypothetical coal stove, called the ‘Future Tech’ stove, which improved the emissions performance of the MCA stoves by the same percentage as the MCA stoves improved upon the traditional stoves and improved indoor concentrations by 20% compared to those in MCA stove homes. Half of all HOB units from baseline were assumed decommissioned by 2024 under Pathway 1, and the other half were assumed retrofitted with cyclone control technologies. All other households were assumed to rely on clean heat from other sources. In addition to the power plant assumptions under BAU, Pathway 1 assumed high efficiency control devices, such as electrostatic precipitators, installed on units CHP-2, CHP-3, and CHP-4. This is a significant upgrade to the existing CHP infrastructure, which includes wet scrubbers or electrostatic precipitators, depending on the facility. For vehicles, the BAU rate of growth was assumed for Pathway 1, but with the implementation of Euro V emissions standards.

Pathway 2, or transition to cleaner fuels and technologies, assumed feasible but ambitious rates of change in all sectors by 2024. Solid fuel combustion was assumed eliminated in households. CHP-3 and CHP-4 were assigned high efficiency control technologies. CHP-2 was decommissioned by 2024 and replaced with renewables and/or imports (i.e. sources with negligible impacts on UB air quality). A 50% reduction in traffic emissions over Pathway 1 was assumed, opportunities for which include but are not limited to higher adoption rates for mass transit use, transportation network enhancements to improve traffic flow, and adoption of Euro VI standards, which include an additional 50% reduction in PM emission rates from heavy duty diesel vehicles compared to Euro V standards.

Throughout BAU and the alternative pathways tobacco smoking prevalence among households (not individuals) was maintained at 45% of households. This figure was based on a series of surveys [22,23] of gers and houses in UB conducted by the Millennium Challenge Corporation during the 2012–2013 winter and corroborated by recent studies of national smoking rates [24,25].

### Population and household numbers

**Fig 2:**
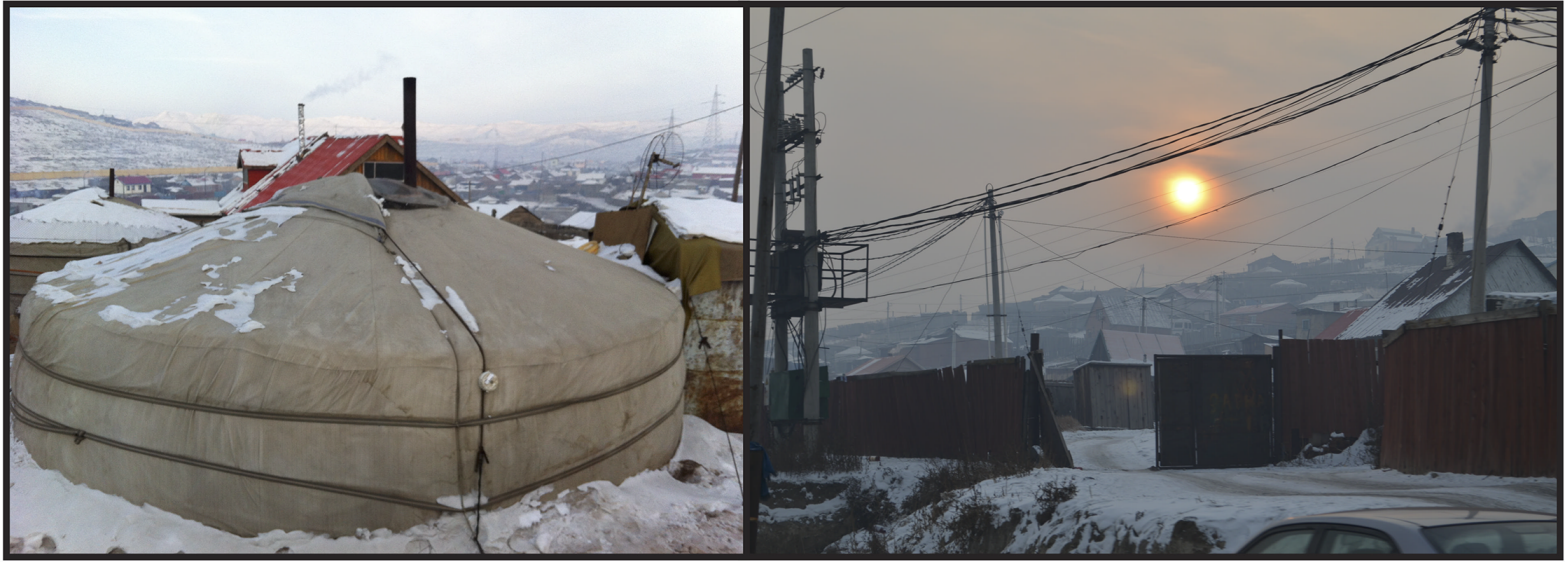
Left, a traditional ger dwelling. right, houses typical of the peri-urban regions of UB. (Credit: L. Drew Hill).

Demographic conditions were estimated for 2006 through 2024. The methods and sources used are described in detail in the supplemental text (S1 Text). Briefly, city-wide population data all residents and, specifically, those < 5 years old were estimated from historical data and National Statistics Office medium growth projections [18]. Detailed information on the spatial distribution of population and household number by household type was obtained from 2012 city census data [17]. Estimates were made for the number, type, and general location of households in UB through 2024 using historical demographic data and official city projections. Household types most relevant to the UB context were identified as gers, single-family houses, and multi-family apartments. Gers in the peri-urban area of UB are circular traditional dwellings with multiple felt layers and a waterproof outer shell covering a wooden lattice frame (Fig 2). Houses in the peri-urban area are often locally constructed wood, cement, or brick structures and, while they vary considerably in layout and construction, are generally in the style of traditional western houses in which one extended family resides. Apartments are identified as buildings within which two or more families are living and, in most cases, are large complexes that house dozens of families.

The 2014 population of UB was estimated at 1,355,176 residents distributed among 86,246 gers, 106,353 houses, and 179,718 apartments. Projections indicated that by 2024 the population would grow nearly 40% and experience a substantial shift to apartment dwelling with over 65% of the population living in multi-family buildings (Table 3). It should be noted that while our stove-number projection technique employed an underlying assumption that each household typically relied on a single stove, some homes may employ more than one stove (e.g. a ‘home’ may consist of two ger, each heated by their own stove) [9]. In stove-heated homes, outdoor models accounted for this by inflating stove estimates and, thus, stove emissions upwards by 20% [9], while the exposure model was applied using a single primary stove type for each household.

**Table 3:** Estimates of age-specific city-wide population and household number by home type, and estimated household size.

Year	Population ^1^		Number of Households ^2^			Pop. per
-	Total Pop.	Pop. 0–4 Years	Ger	House	Apartment	Household ^2^
2014	1,355,176	148,219	86,246	106,353	179,718	3.64
2015	1,407,196	155,551	88,547	109,191	197,539	3.56
2016	1,459,516	158,438	90,684	111,826	216,586	3.48
2017	1,511,836	161,325	92,616	114,209	236,854	3.41
2018	1,564,157	164,212	94,323	116,313	258,369	3.34
2019	1,616,477	167,099	95,781	118,112	281,152	3.27
2020	1,668,797	169,986	96,967	119,574	305,219	3.20
2021	1,715,748	168,427	96,997	119,611	330,782	3.13
2022	1,762,700	166,869	96,667	119,204	357,645	3.07
2023	1,809,651	165,310	95,954	118,324	385,792	3.02
2024	1,856,603	163,752	94,834	116,943	415,195	2.96

1.Interpolated from five-year “medium growth” (version 1b) projections identified in the 2010 Population and Housing Census of Mongolia Report [18].

2.Estimated using the techniques and sources described in S1 Text.

### Outdoor ambient air quality modeling

Air quality modeling was conducted to estimate outdoor PM_2.5_ mass concentrations. The modeling methodology followed that used by Social Impact for an impact evaluation of the Energy-Efficient Stove Subsidy Program of the Millennium Challenge Mongolia Energy and Environment Project (MCA impact evaluation), detailed in the full report [9]. Emission categories were expanded to include LPB, HOB, motor vehicles, and Combined Heat & Power plants (CHP) in addition to residential heating stoves. Sources not included in the model were heating stoves in kiosks, industrial emissions including kilns, re-suspended road dust, and windblown dust.

#### Emissions data

Table 4 summarizes total annual emissions from the major sources considered at baseline and in 2024 under BAU the two alternative development pathways.

**Table 4:** Estimated annual PM_2.5_ emissions from major sources (tons/yr).

Pathway	Vehicles	Power Plants	Heat Only Boilers	Household Stoves & LPB
2014 Baseline	384	11,500	1,300	1,700
2024 BAU	500	12,000	1,300	1,900
2024 Pathway 1	96	1,900	390	640
2024 Pathway 2	48	1,830	0	0

Residential heating stove emissions were assumed zero during the summer period, April through September. An MCA stove emissions profile was taken from values reported in the MCA impact evaluation as weighted by the sales-based prevalence of three variations of the MCA stove (Ulzii, Khas, and Dul) detailed in the same publication [9]. Data on the emissions profiles of low-pressure boilers and semi-coke coal stoves in UB were unavailable, and so they were conservatively assigned the emissions profiles of MCA stoves. “Future Tech” stove outdoor emissions were assigned by applying to the MCA emissions profile the same ~ 60% reduction estimated for the transition from traditional coal stoves to MCA stoves during the Social Impact evaluation [9]. HOB emissions were informed by a PM_10_ HOB emissions inventory prepared by the Japanese International Cooperation Agency (JICA) [19]. All HOB stack emissions were assumed to be in the PM_2.5_ size range. These inventories were used with no modifications for the baseline (2014) and BAU (2024) PM_2.5_ emission inventories. Pathway 1 assumed that in 2024, an overall 70% reduction in HOB emissions would be reached, which was consistent with decommissioning 50% of HOB and adopting high efficiency cyclones as a control strategy on all others [19]. Pathway 2 assumed all HOBs would be decommissioned by 2024.

Baseline power plant emissions were taken from a recent JICA [19] PM_10_ emission inventory for CHP-2, CHP-3, and CHP-4. All stack emissions were assumed to be in the PM_2.5_ size range. CHP-5 was assumed to meet the U.S. New Source Performance Standards for electric utility power plants, which is 0.015 lb PM/MMBtu [26], and have an electricity generation rate of 1870 kWh/ton coal and a coal heat content of 19.53 MMBtu/ton. Assuming the plant would operate continuously throughout the year, the estimated PM emissions were 511 tons/year, all of which were assumed to be PM_2.5_. In Pathway 1, the PM capture rate for the high efficiency control devices installed on units CHP-2, CHP-3, and CHP-4 was conservatively assumed at 98%. The 98% capture rate was applied to an assumed uncontrolled emission factor of 16.6 kg PM/ton coal. The renewable and imported generation capacity in Pathway 2 assumed no emissions-related impacts on UB.

A simple scaling approach as previously described was used for motor vehicle emissions pathways, and did not account for changes in the fleet composition over time as insufficient details for the JICA 2010 inventory were available to make more sophisticated projections. All exhaust emissions were assumed to be in the PM_2.5_ size range. For diesel vehicles the Euro V PM emission standards are 80% -93% lower than the Euro III standards depending on vehicle class. There were no Euro standards for PM emissions from gasoline-fueled vehicles and thus 90% overall reduction would not be realized between BAU and Pathway 1. However, gasoline vehicle Total Hydrocarbon standards are 50% lower for Euro V compared to Euro III. This could result in some PM reductions for the cold wintertime conditions, which favor semi-volatile gaseous compounds entering the particle phase. Overall, Pathway 1 employed a 75% reduction in vehicle emissions in 2024 compared to those at baseline. Pathway 2 assumed a simple 50% reduction in traffic emissions over Pathway 1, as previously discussed.

#### Model techniques

Existing power plants were modeled as point sources using available geographic location and stack properties data [19]. Residential heating stoves, HOB, and motor vehicle emissions were modeled as area sources. The greater UB region was discretized into 6,298 grid cells, each with dimension 1 km × 1 km. Emissions were allocated to these grid cells and the center of each grid cell was used as a receptor site for which modeled PM_**2.5**_ concentrations were generated. Modeling was conducted at hourly resolution using the ISCST3 Gaussian dispersion model [27,28] with pre- and post-processing using GIS [29]. Two satellite districts–Baganuur and Bagakhangai–were excluded from modeling. These districts are remote, low population zones that are not contiguous with UB’s other districts. No HOB or LPB emission sources were assigned to these districts; all previously described HOB and LPB sources were included in the modeled districts only. Seasonal-average PM_**2.5**_ concentrations for these districts, which together account for less than 3% of all households, are assumed to be at the 10^**th**^ percentile (decile) of population-weighted PM_**2.5**_ concentration distributions for the remaining districts that were modeled.

Estimation of residential heating stove emissions and allocation of these emissions in space and time followed approaches developed for the Social Impact MCA impact evaluation [9]. The baseline pathway treated all stoves as MCA stoves, which were increased proportionally to adjust for population growth to 2024. Stoves emissions were modeled as area sources corresponding to the 1 km × 1 km grids.

Motor vehicle and HOB emissions were spatially allocated using emissions fields from 2010 with a resolution of 0.01°× 0.01° [8] that were re-projected in GIS by contouring the data and then calculating area weighted means for the 1 km × 1 km grids used for the modeling. HOB emissions were temporally allocated using weights employed in a 2013 examination of particulate pollution in UB [8]. Motor vehicle emissions were held constant for each season and were allocated to hour of day using a typical urban profile with morning and afternoon rush hour peaks.

Year 2012 population by dwelling type (ger, house, and apartment) at the level of Khoroo, or Mongolian administrative sub-division similar to a sub-district, was also allocated to the 1 km × 1 km grids using area weighted sums. Projected changes in the peri-urban population between 2012 and the baseline and 2024 BAU and alternative pathway years were distributed across grid cells in proportion to the 2012 peri-urban population for both gers and houses. Projected changes in the population residing in apartments were allocated in proportion to the total population in each grid.

Air quality modeling was conducted at hourly resolution using meteorology data from April 2012 through March 2013 provided to us by the National Agency for Meteorology, Hydrology, and Environmental Monitoring of Mongolia–data available to other users upon written request to the Environmental Monitoring Department at what is now the National Agency for Meteorology and Environmental Monitoring of Mongolia. Un-modeled emission sources were assumed to have a spatially and temporally constant contribution of 10 μg/m^3^ across the city and over the ten-year assessment period. The model underestimated outdoor PM_2.5_ measurements conducted during the 2012–2013 winter heating season and these measurement data [9,22,23] were used to calibrate the model. The hourly modeled concentration fields were used to construct daytime (8:00–18:00) and nighttime 18:00–8:00) average concentrations for summer (April through September) and winter (October through March). These gridded concentration estimates were combined with the gridded population data to estimate citywide population-weighted outdoor PM_2.5_ concentrations by home type (ger, house, apartment). Further detail about the modeling and calibration are provided in S1 Text.

### Indoor air quality estimates

The vast majority of gers and houses in peri-urban areas heat with raw coal lit by small amounts of wood in small chimney stoves, while apartment households almost exclusively employ CIU heat that creates no indoor emissions. These differences combined with variations in outdoor particle infiltration between building types likely result in substantially different indoor concentrations between gers, houses, and apartments. Indoor concentrations of PM_2.5_ were thus estimated by home type, household heating source, presence of secondhand tobacco smoke (SHS), and season. Estimates were made for 2014 (baseline) and 2024 under BAU and the two alternative policy pathways.

Indoor air concentrations in homes with heating stoves were estimated by applying linear modeling techniques to data collected during the 2012–2013 winter season discussed in [9] and accessible freely online [22,23]. This model was designed to account for the impacts of stove type, home type, and household tobacco smoking status on household PM_2.5_ concentrations while controlling for when the samples were taken. Methods and results are described in more detail in S1 Text. Wintertime indoor concentrations in homes with “Future Tech” stoves (Pathway 1) were assumed 20% lower than those found in MCA stove homes based on the assumption that such a stove would be designed to reduce indoor fugitive emissions. Wintertime indoor concentrations in homes with LPB and semi-coke stoves were assigned those of homes with MCA stoves, in accordance with the conservative emissions assignments for these stove types as discussed above.

Indoor concentrations in homes that employ CIU heating sources like district heating, HOB, electric heat, or gas-based heat were estimated differently from those with heating stoves. Such concentrations were assumed to be governed primarily by SHS and by the penetration of outdoor PM_2.5_ into the indoor environment. Infiltration efficiencies were estimated at 64% in the summer and 53% in the winter for houses and apartments, and 100% in the summer and 70% in the winter for gers based on blower door tests and relevant literature detailed in S1 Text. Indoor concentrations in households with clean heating were estimated by linearly applying infiltration efficiencies to home-type specific population-weighted outdoor ambient concentrations.

Smoking rates in Mongolia are among the highest in the world [24,25]. As previously discussed, SHS was assumed present in 45 percent of households. Recent nation-wide bans on public indoor smoking [30] suggest indoor SHS may only make considerable contributions to exposure in personal, private indoor environments. Indoor concentration estimates thereby conservatively assume SHS occurred only indoors at home and thus contributions from SHS were applied only to nighttime indoor concentration estimates.

For simplicity and due to limited information on Mongolian workplace environments, the concentration profiles of the indoor environments in which the population spends their time away from home were assumed the same as those of their home indoor environments.

### Time activity

Time activity information was informed by a recent survey of UB households [22,23] as well as an understanding of the local job market and commuting patterns [31]. It is expected that small children spend more time indoors than the rest of the population, and so time activity was calculated separately for children (< 5 years old) and non-children (> 5 years old). Because the social pension fund provides a homecare allowance to mothers in UB with children less than 2 years old [4] and because day care and nursery are not financially accessible to many households, it is assumed that a subset of the non-child population is charged with taking care of the children, and so every child < 5 years old was assigned one non-child as a “caretaker”. Time spent by residents in each microenvironment likely differs by socio-demographics and season, but because no data on this breakdown were available, we conservatively assumed that the average day for “non-children” would be spent 75% indoors and 25% outdoors, the average day for children and caretakers would be spent approximately 100% indoors, and the average nighttime for all residents would be spent approximately 100% indoors.

### Exposure estimation

The UB population was divided into sub-groups based on the major exposure-related features of the indoor model and time activity estimates: home type, heating type, presence of SHS at home, and age. More specifically, sub-groups were made for children (< 5 years old), caretakers (≥ 5 years, assumed 1 per child), and all others (≥ 5 years) in smoking and non-smoking households representing each of the following home-heating combinations: gers with MCA stoves or semi-coke coal stoves, future tech stoves, or clean heat; houses with MCA stoves or semi-coke coal stoves or low pressure boilers, future tech stoves, or clean heat; and apartments with clean heat. For exposure estimation, children, caretakers, and non-children were distributed evenly to each household, and exposures were not distinguished by gender. Baganuur and Bagakhangai—the two districts for which ambient air quality estimates were handled outside of the outdoor models—were assigned the same distribution of population sub-groups as the overall population, with the exception that none of the LPB homes were included in these excluded districts as previously discussed. Household proportions in Baganuur and Bagakhangai were identified in the 2012 city census [17] as comprising 3.0%, 2.1%, and 3.4% of UB’s total apartment, house, and ger households, respectively. These proportions were assumed constant through 2024. Population sub-groups totaled 18 at baseline and in 2024 under BAU and Pathway 2. Pathway 1 included 30 sub-groups due to the presence of additional heating types.

Average annual PM_2.5_ exposure concentrations for each population sub-group “i” were estimated at baseline and in 2024 under each pathway “j” (BAU, Pathway 1, or Pathway 2) by averaging seasonal exposure values (_S_; winter as April–September, summer as October–March) calculated from indoor (_in_) and outdoor (_out_) concentrations (C) at night (_N_; 18:00–8:00) and during the day (_D_; 8:00–18:00) as weighted by the fraction of time (t) during a typical 24-hour period spent in each environment during the specified time period (Eq 1).

$${\begin{array}{c} Annual\\ Average\;Exposure \end{array}}_{i,j}=\sum_{s=summer}^{s=winter}{\left(\frac{1}{2}∙(\begin{array}{c} (t_{in,D,i}∙C_{in,D,i})+(t_{out,D,i}∙C_{out,D,i})+\\ \left({t_{in,N,i}∙C}_{in,N,i}\right) \end{array})\right)}_{S}$$ (1)

Citywide population-weighted average exposures were calculated by aggregating the exposure concentrations of each sub-group from i = 1 to i =“n”, where n is the total number of sub-groups in each pathway-year j, as weighted by their representative fraction (λ) of the total population (Eq 2).

$${\begin{array}{c} Pop.\;Weighted\;Annual\;\\ Average\;Exposure \end{array}}_{j}=\sum_{i=1}^{n}\left(\lambda_{i,j}∙{\begin{array}{c} Annual\;\\ Average\;Exposure \end{array}}_{i,j}\right)$$ (2)

### Estimating health effects

Burden attributable to PM_2.5_ exposures was calculated for lung cancer, ischemic heart disease (IHD), stroke, and chronic obstructive pulmonary disorder (COPD) in all UB residents as well as acute lower respiratory tract infection (ALRI) in children (ages 0–4 years) for 2014–2024 using a version of the Household Air Pollution Intervention Tool (HAPIT) [15] that was modified to accommodate UB-specific background data and projections through 2024. Disease-specific background mortality for the capital city was projected through 2024 using historical data for 2006–2012 provided by the Health Development Center of the Ministry of Health and Sports in conjunction with the Mongolian National University of Medical Sciences. Deaths were obtained for 2006–2012 that matched the ICD-10 codes used in the 2010 GBD [32] to estimate illness from PM_2.5_. Mortality projections were adjusted to better agree with national estimates produced by the Institute for Health Metrics and Evaluation [12]. The methods and results are described further in S1 Text.

Average annual exposures were used to calculate disease-specific relative risks (RR) of mortality due to PM_2.5_ exposure in each population sub-group. Mean, lower bound, and upper bound RR were taken from the integrated exposure-response functions produced by Burnett et al [33] and applied using a counterfactual exposure of 12.0 μg/m^3^ (i.e. RR = 1 at 12.0 μg/m^3^). This counterfactual represents the US Environmental Protection Agency’s annual for PM_2.5_, which is the strictest national PM_2.5_ standard in the world. Mean, lower, and upper estimates of disease-specific RR for each sub-group “i” as well as the proportion of the population that each sub-group represents, “P”, were then applied to Eq 3 [12] to produce a population-wide estimate of the fraction of background mortality from each disease, “k”, attributable to air pollution exposure (population attributable fraction, or PAF).

$${PAF}_{j,k}=\frac{\sum_{i=1}^{n}P_{i,j}∙({RR}_{i,j,k}-1)}{\sum_{i=1}^{n}P_{i,j}∙({RR}_{i,j,k}-1)+1}$$ (3)

Disease-specific PAF estimates for 2015–2023 were linearly interpolated from baseline and 2024 PAF values under BAU and each alternative pathway. Finally, PAF values were applied to estimated background mortality estimates to produce disease-specific estimates of total attributable death in each year. Morbidity was calculated in the form of disability adjusted life years (DALYs). DALYs are widely used to take into account both the age distribution of premature mortality and the severity of non-fatal diseases. Disease-specific DALY estimates were calculated using the national disease-specific Death: DALY ratio produced during the 2010 GBD [12]. The ratios were assumed constant throughout the projection period. The modified HAPIT used in this analysis did not include considerations for exposure cessation lag [34,35]; the estimated health impacts of exposure were assumed to be immediately incurred for simplicity of interpretation. Health burden was calculated not only for BAU and each alternative pathway (“accrued”) but also in terms of benefit of each alternative pathway over BAU exposure levels (“averted”).

## Results

### Outdoor ambient air quality models

**Fig 3:**
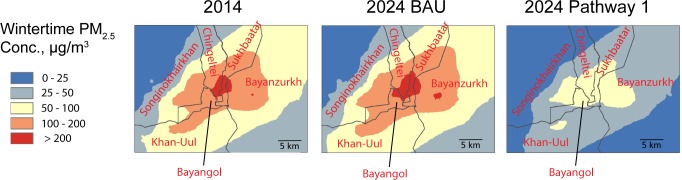
Winter average outdoor PM_2.5_ concentrations for baseline and 2024 under BAU and Pathway 1.

**Fig 4:**
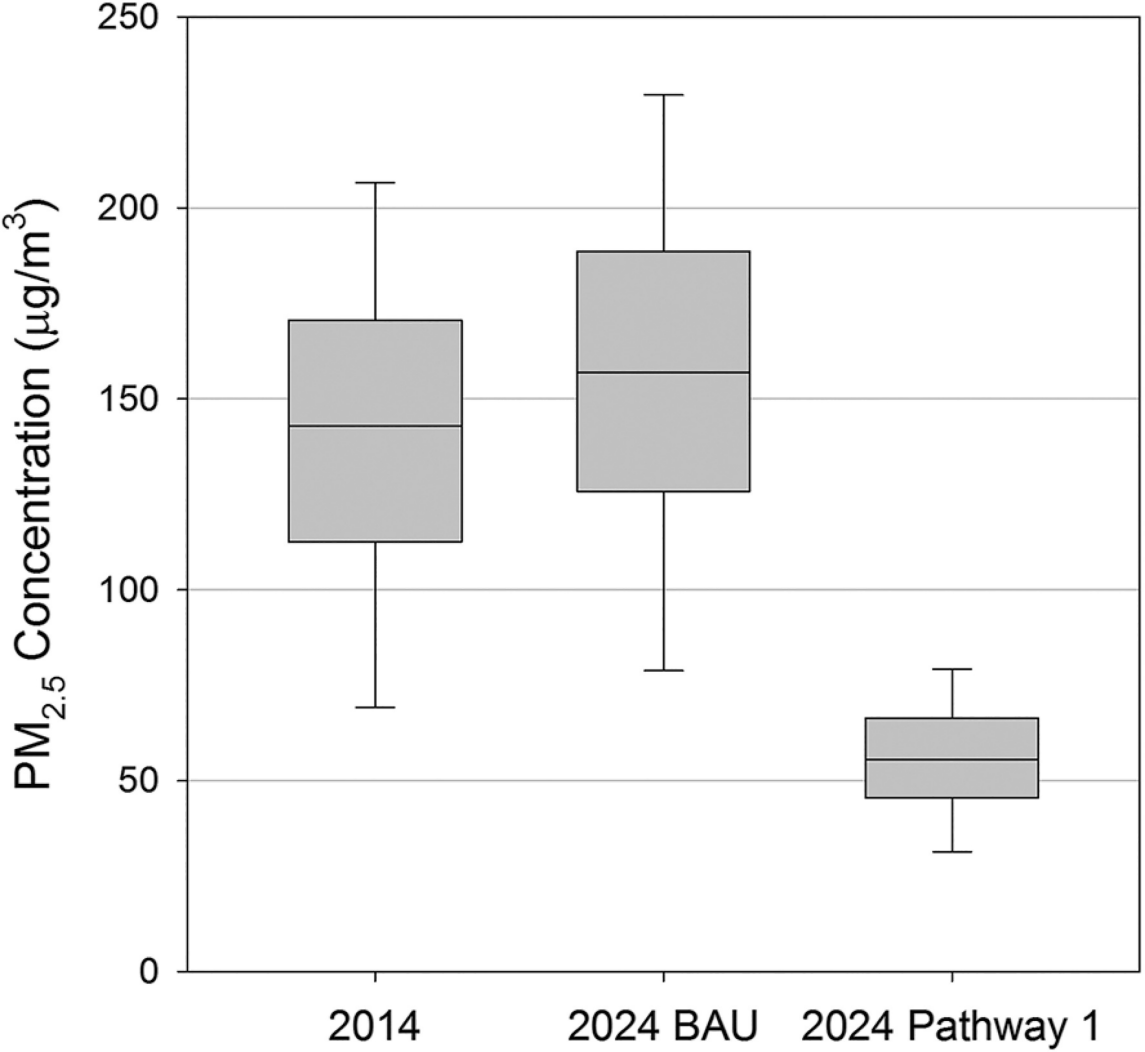
Population weighted wintertime outdoor PM_2.5_ concentrations. Whiskers represent 10^th^ and 90^th^ percentile concentrations.

Fig 3 shows modeled wintertime (October-March) average outdoor PM_2.5_ concentrations for BAU and the alternative pathways. Pathway 2 is excluded because the highest average concentration attributed to the modeled sources was ~2 μg/m^3^ (12 μg/m^3^ when accounting for non-modeled sources). For the panels shown in Fig 3, the model predicts large variations in PM_2.5_ mass concentrations across UB with highest concentrations in the ger areas where residential stoves and HOBs have the largest impact. Given these large spatial variations, outdoor concentration levels between baseline, 2024 BAU, 2024 Pathway 1, and 2024 Pathway 2 are compared using population-weighted measures. Table 5 presents the population-weighted mean outdoor PM_2.5_ for modeled districts. Pathway 1 reduces 2024 wintertime population mean concentration by 65% compared to the 2024 BAU pathway, but the wintertime mean concentration value of 55 μg/m^3^ is still quite high. Fig 4 shows box plots for the population-weighted distribution of wintertime outdoor concentrations. For BAU and each of the alternative policy pathways, 10% of the population resides in areas with PM_2.5_ outdoor concentrations ~50% higher than the mean pathway-specific outdoor concentration reported in Table 5.

**Table 5:** Modeled population weighted mean PM_2.5_ outdoor concentrations (μg/m^3^) by season and year.

Pathway	Summer	Winter			
	Total Pop.	Total Pop.	Ger Pop.	House Pop.	Apt. Pop.
**2014**	16	141	140	148	137
**2024 BAU**	19	156	154	163	154
**2024 Pathway 1**	11	55	55	58	55
**2024 Pathway 2**	11	11	11	11	12

### Indoor air quality estimates

Average wintertime indoor concentrations for non-smoking homes heating with MCA stoves, LPB (houses only), and stoves using semi-coke coal were modeled at 107.0 μg/m^3^ for gers and 118.3 μg/m^3^ for houses. Wintertime indoor concentrations for homes with Future Tech heating stoves were assigned at 20% lower than homes with MCA stoves. Population-weighted indoor wintertime concentrations for non-smoking homes with clean heating at baseline and in 2024 are shown in Table 6. Estimates of population-weighted summertime indoor concentrations in non-smoking homes at baseline and in 2024 are shown in Table 7. Based on the results of the indoor concentration model, a contribution from SHS of 18.1 μg/m^3^ in gers and 20.0 μg/m^3^ in houses and apartments was applied to the nighttime indoor concentration estimates of smoking households.

**Table 6:** Estimated average wintertime indoor PM_2.5_ concentrations for non-smoking homes with CIU heat, by home type and year.

	2014	2024 BAU	2024 Pathway 1	2024 Pathway 2
*Ger*	98	108	39	8
*House*	79	86	31	6
*Apartment*	73	82	29	6

**Table 7:** Estimated average summertime indoor PM_2.5_ concentrations for all non-smoking homes, by home type and year.

	2014	2024 BAU	2024 Pathway 1	2024 Pathway 2
*Ger*	15	17	11	11
*House*	10	11	7	7
*Apartment*	11	13	7	7

### Exposure

**Fig 5:**
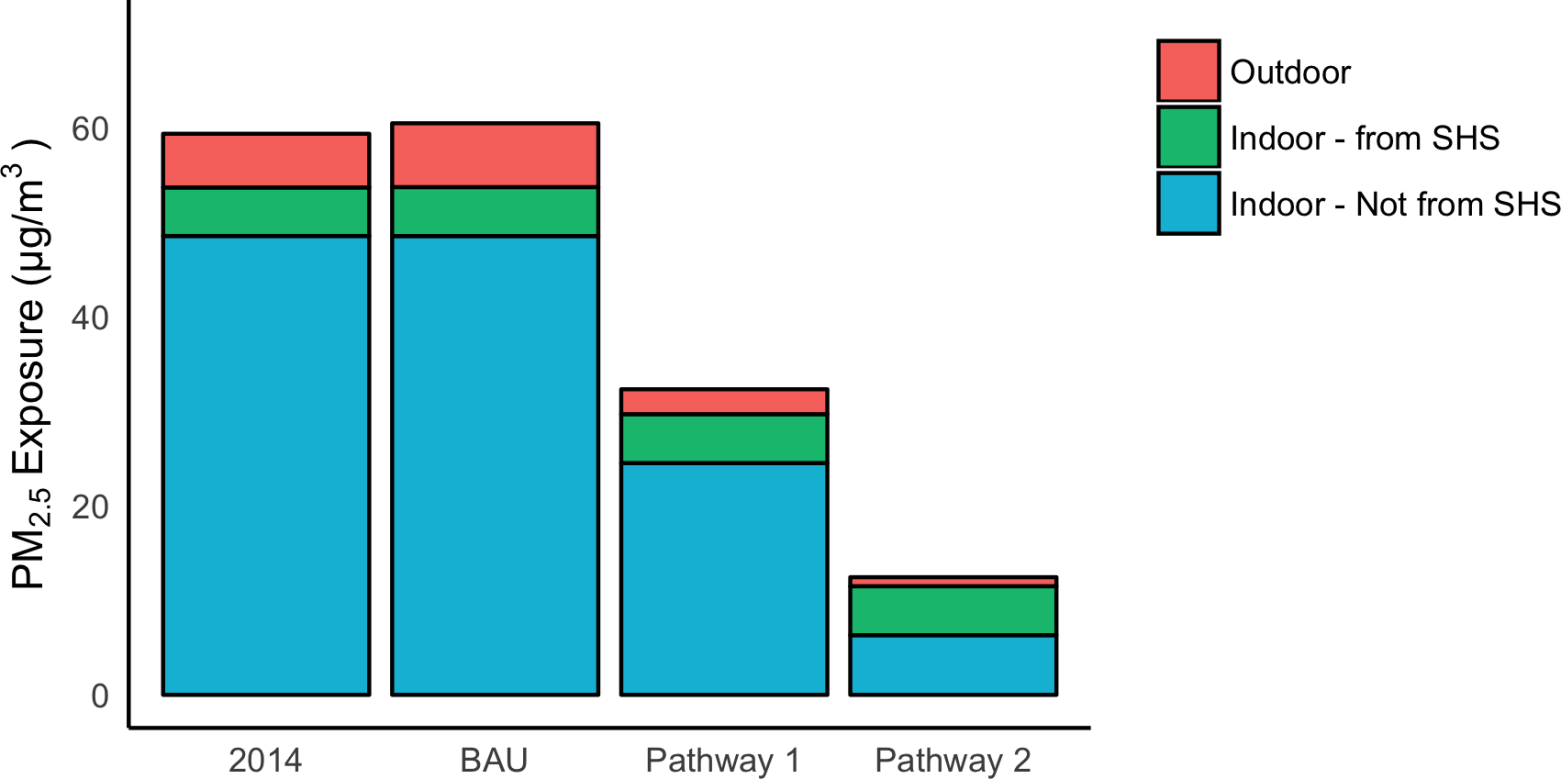
Exposures in 2014 and 2024 under BAU and alternative policy pathways, by environment. Indoor exposures are stratified by SHS and non-SHS environments. The difference between indoor and outdoor contribution to total exposure is primarily from the disproportionately high fraction of time spent indoors.

Table 8 shows population-weighted average exposure estimates of 59 μg/m^3^ in 2014, and 60 μg/m^3^, 32 μg/m^3^, and 12 μg/m^3^ in 2024 under BAU, Pathway 1, and Pathway 2, respectively, with the greatest exposures consistently affecting ger-dwelling residents. A continuation of current policy trends (BAU) slightly increased population exposures by 2024. In contrast, the modest control measures of Pathway 1 reduced exposures by 45% compared to 2014 levels. The shift to clean technologies in Pathway 2 reduced population exposures by 80%. With the exception of Pathway 2, wintertime exposures in gers and houses dominated city-wide average exposures. Summertime concentrations varied only modestly across BAU and the alternative pathways. Fig 5 shows the relative contributions from exposures experienced indoors and outdoors. Exposures incurred indoors accounted for most of the annual averages with a large portion of this resulting from SHS, especially in Pathways 1 and 2. Substantial differences in average annual exposures were seen between home types, with those in houses and gers receiving the highest exposures, and those in apartments on average about 30% lower.

**Table 8:** Estimated population-weighted average exposures by home type, year, and season (μg/m^3^).

	Annual				Winter					Summer			
	2014	2024 BAU	2024 Path 1	2024 Path 2	2014	2024 BAU	2024 Path 1	2024 Path 2	2014		2024 BAU	2024 Path 1	2024 Path 2
*Ger*	66	68	52	14	113	114	87	13	20		22	16	15
*House*	70	71	44	12	125	126	76	12	15		17	13	12
*Apartment*	50	56	25	12	83	93	36	12	17		19	13	13
**All Population**	**59**	**60**	**32**	**12**	**102**	**102**	**51**	**12**	**17**		**19**	**13**	**13**

### Health impacts

**Fig 6:**
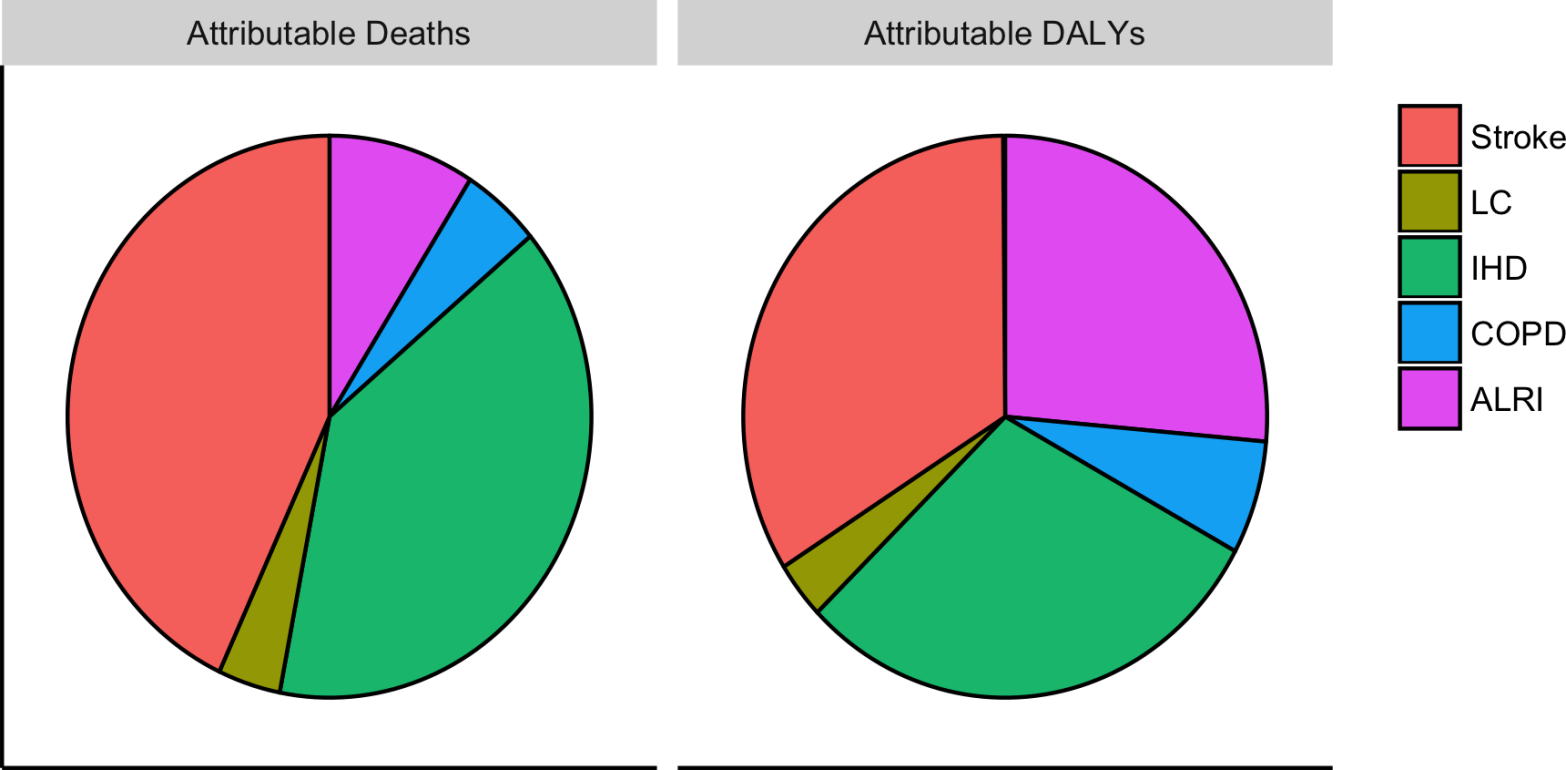
Distribution by disease of burden attributable to air pollution in UB at baseline. Note the higher importance for ALRI in the DALY distribution because it affects young children.

**Fig 7:**
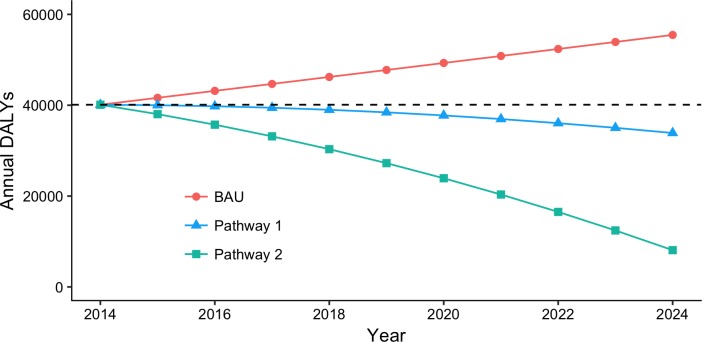
Estimated disease burden of PM_2.5_ over the assessment period for BAU and each alternative pathway. Burden of measure is annual DALYs, or DALYs/year. Baseline value (2014) is marked as a dashed line. Pathway 1 averts about 110,000 total DALYs from BAU policies. The stronger reduction measures of Pathway 2 avert about 240,000 total DALYs.

**Fig 8:**
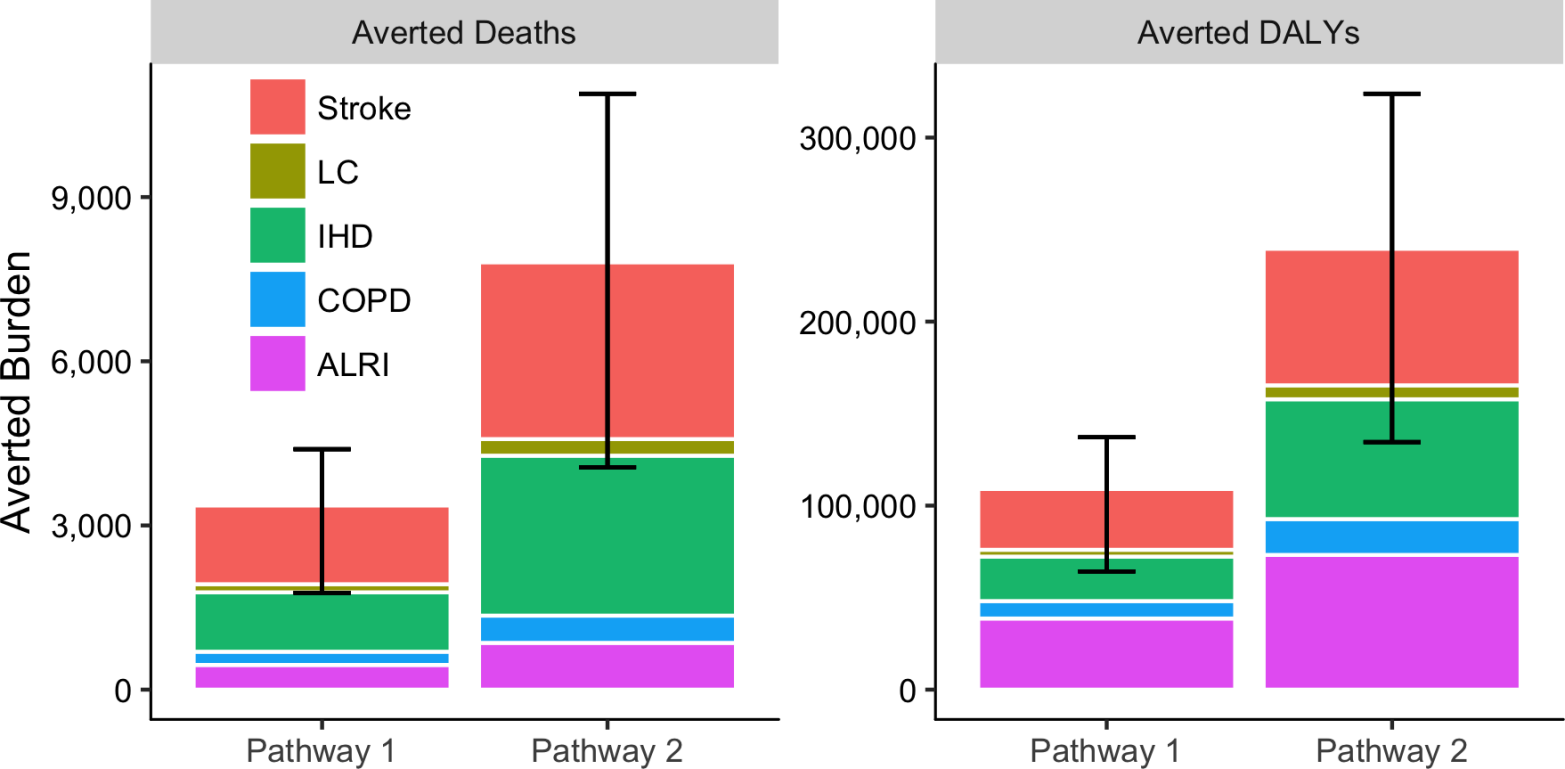
Estimated burden averted from BAU by measures taken in Pathways 1 and 2 (2014–2024). Pathway 2 would save more lives than Pathway 1 by more than a factor of 2. Lower and upper bounds on total values are shown as whiskers. Note the greater importance of child ALRI in averted DALYs as compared to averted deaths.

We estimated exposure to PM_2.5_ in UB in 2014 to be responsible for 33% (lower: 23%, upper: 42%) of all ALRI deaths in children, 19% (lower: 9%, upper: 28%) of all COPD deaths, 27% (lower: 19%, upper: 42%) of all IHD deaths, 24% (lower: 8%, upper: 34%) of all lung cancer deaths, and 42% (lower: 14%, upper: 54%) of all stroke deaths, for a total of 1,400 attributable deaths (lower: 710, upper: 1,900) and 40,000 attributable DALYs (lower: 22,000, upper: 55,000) (Table 9). Deaths and DALYs attributable to PM_2.5_ at baseline were dominated by cardiovascular disease (Fig 6). This pattern was consistent throughout the analysis period. Child ALRI comprised about 25% of attributable morbidity when totaled over the entire 2014–2024 period under each pathway. Over the period 2014–2024, an estimated 18,000 attributable deaths (lower: 9,300, upper: 25,000) and 530,000 attributable DALYs (lower: 290,000, upper: 720,000) were accrued under a BAU pathway. Fig 7 shows that these values were reduced by about 20% under Pathway 1, and about 45% under Pathway 2. Nearly all of the deaths averted by Pathways 1 and 2 resulted from IHD and stroke (Fig 8). Averted DALY’s in both alternative pathways were dominated by child ALRI, IHD, and stroke.

**Table 9:** Excess deaths and DALYs attributable to PM_2.5_, BAU, Pathway 1, & Pathway 2 (per 1000 capita values rounded to two significant digits).

* *	Accrued, 2014–2024 (per avg. 1000 Capita)	Lower, Upper Bounds	Incurred in 2014 (per 1000 Capita)	Lower, Upper Bounds	Incurred in Final Year of BAU or Pathway, 2024 (per 1000 Capita)	Lower, Upper Bounds
*Deaths*						
*BAU*	18,000 (11)	9,300–25,000			1,800 (0.99)	980–2,600
*Pathway 1*	14,000 (9.0)	7,500–20,000	1,400 (1.0)	710–1,900	1,200 (0.63)	630–1,700
*Pathway 2*	9,800 (6.4)	5,200–14,000			310 (0.16)	180–450
*DALYs*						
*BAU*	530,000 (330)	290,000–720,000			55,000 (30)	31,000–77,000
*Pathway 1*	420,000 (260)	230,000–590,000	40,000 (30)	22,000–55,000	34,000 (18)	18,000–49,000
*Pathway 2*	290,000 (190)	160,000–400,000			8,100 (4.4)	4,400–12,000

**Fig 9:**
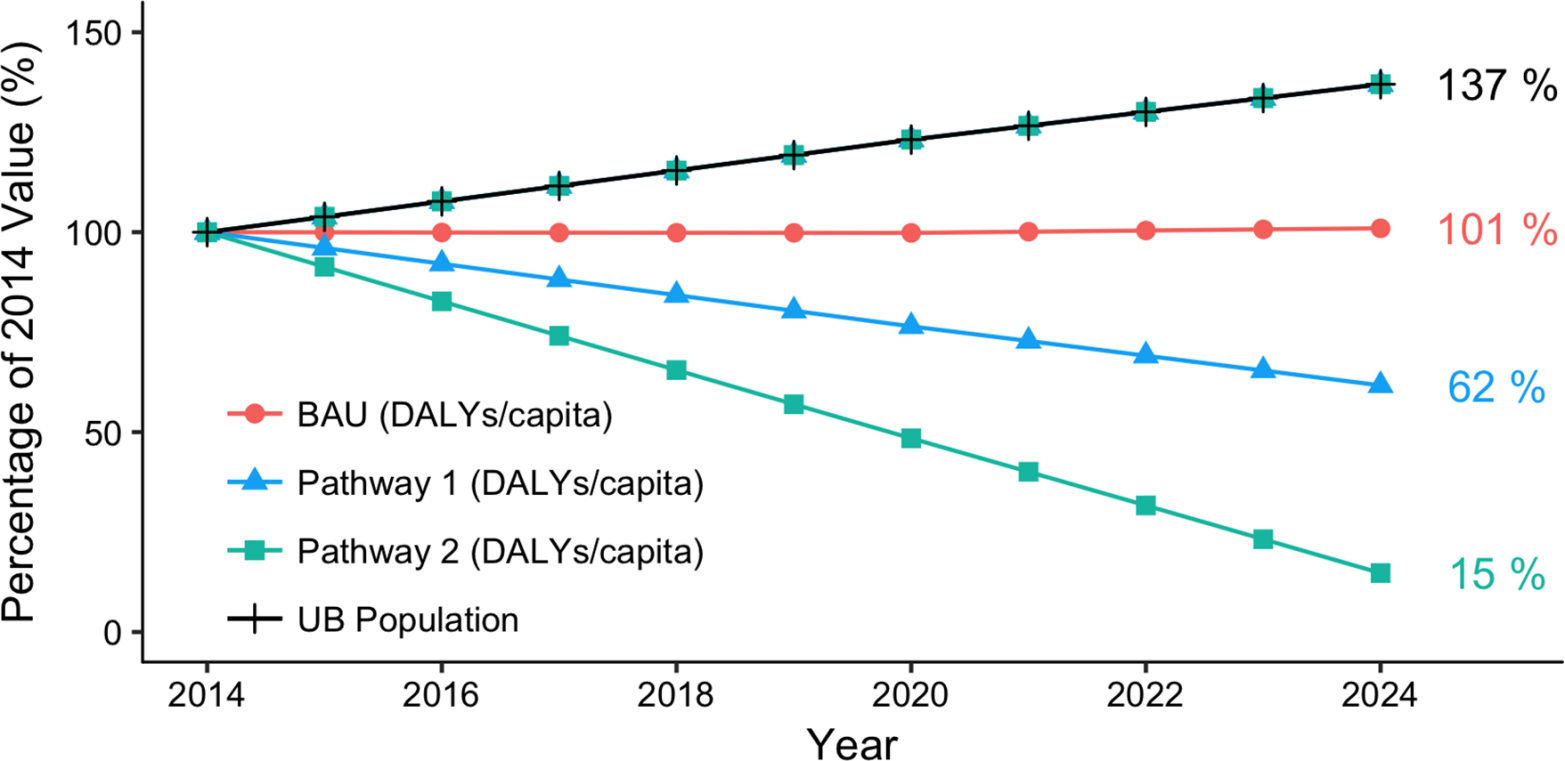
Relative projected health impacts per capita for BAU and alternative pathways (2014–2024). Relative projected urban population is also shown. Note that 2014 values are set at 100%. Pathway 2 would reduce impacts to near-counterfactual levels by 2024.

The prolonged reduction period resulted in about 9,800 (lower: 5,200, upper: 14,000) unavoidable deaths and 290,000 (lower: 160,000, upper: 400,000) unavoidable DALYs under the most rigorous reduction pathway between 2014–2024 (Fig 8), but annual reductions were substantial. In 2024, an estimated 1,800 (lower: 980, upper: 2,600) deaths and 55,000 (lower: 31,000, upper: 77,000) DALYs were still incurred under BAU. This was reduced by about 35% under Pathway 1 and about 85% under Pathway 2 (Table 9). Ulaanbaatar’s rapidly increasing population was accounted for by an examination of annual per capita burden (Table 9 and Fig 9). Changes in annual per-capita burden between baseline and 2024 under all pathways were similar to those estimated for total burden.

## Discussion

Modeled average annual exposures in Ulaanbaatar (estimated at 59 μg/m^3^ in 2014) remained high, despite a wide range of pollution reduction measures recently enacted by the Mongolian government, including ambient air quality standards [36], energy efficiency programs [9], anti-smoking laws [30], and improved stove subsidies [9]. Our models indicated that this trend was driven primarily by high wintertime indoor concentrations influenced substantially by the infiltration of outdoor pollution. In gers and houses heating with LPB (houses only), MCA stoves, and stoves using semi-coke coal, modeled wintertime indoor concentrations were more than ten times higher than the WHO PM_2.5_ annual Air Quality Guideline (10 μg/m^3^) [37], a recommendation considered necessary to be fully health protective. For regions with very high concentrations, a more reasonable context for indoor concentration comparisons may be the WHO Air Quality Guideline interim targets, which are designed as achievable incremental policy goals. Even still, modeled indoor concentrations in gers and houses heating with LPB (houses only), MCA stoves, and stoves using semi-coke coal are more than three times higher than the highest interim target (35 μg/m3).

We estimated that PM_2.5_ exposures in 2014 were responsible for 33% (lower: 23%, upper: 42%) of all deaths from ALRI in children and 19% (lower: 9%, upper: 28%), 27% (lower: 19%, upper: 42%), 24% (lower: 8%, upper: 34%), and 42% (lower: 14%, upper: 54%) of all deaths from COPD, IHD, lung cancer, and stroke, respectively. PM_2.5_ related mortality at baseline and as accrued under all pathways was driven by cardiovascular disease, while attributable morbidity was more evenly distributed between IHD, Stroke, and ALRI in children (< 5 years). These estimates and trends were consistent with global and national estimates from the GBD [11], and identified children as a local population of particular vulnerability. They were also within the range of citywide findings from Allen et al. [3], which were based on earlier exposure-response functions, different disease groupings, a lower counterfactual of 7.5 μg/m^3^, and outdoor concentrations alone.

A business as usual approach to energy policies in UB will have little impact on citywide PM_2.5_ exposures by 2024 yet may result in a substantial increase in total health burden because of large increases in projected urban population. A package of policies targeting reductions in both indoor and outdoor emissions from household coal stoves alongside aggressive improvements in power and traffic sectors could reduce annual average population-weighted PM_2.5_ exposures by nearly 80% and annual per-capita attributable health burden by about 85% by 2024. A package of more moderate emissions control policies, including cleaner-burning coal stoves and modest improvements to the city’s power plants and vehicle fleets, may reduce PM_2.5_ exposures by 45% but would have less effect on health burden due in part to the non-linearity of the relationship between PM_2.5_ exposure and risk for many diseases [33]. When energy-related emissions are ultimately reduced, environmental tobacco will play an important role in local disease burden if not aggressively targeted by regulators.

Our investigation builds upon a small but growing body of air quality research in Mongolia [3,6,8,10,38–42] and is the first to both examine and predict population-wide PM_2.5_ exposures in UB that are integrated across environments and account for contributions from SHS. We are aware of two studies that have directly measured personal PM_2.5_ exposures in individuals in UB, both of which are difficult to interpret within the context of our analysis and so were not explored in depth. One of these studies [42] reported primarily on peak concentrations and is thus difficult to compare with our estimates of longer term averages. The other study [41] examined personal PM_2.5_ exposures between 9:00 and 17:00 in hospital patients in March and July. However, that study reported only on “indoor” and “outdoor” personal exposure levels, the definitions of which are unclear, did not disclose a sample size, and did not distinguish exposure statistics by relevant demographics like heating type or smoking status.

Most inferences about the population health impacts of PM_2.5_ in UB and greater Mongolia have relied on outdoor concentrations modeled from emissions, chemical transport estimates, and/or measurements taken from a small number of outdoor, fixed-site monitors [3,6,8] or outdoor concentrations measured using hybrid satellite techniques [13]. But satellite estimates have been found particularly unreliable in UB because of poor resolution in winter and at night, when concentrations are highest in the region [43,44], and evidence suggests that intra-urban outdoor air quality is highly heterogeneous and that city-wide outdoor estimates rooted in data from a small number of sites may lead to considerable error when applied to health impact evaluation [45]. Our approach included outdoor modeling techniques, but tempered issues of exposure misclassification by incorporating indoor concentrations weighted by locally relevant and age-specific time activity estimates.

A 2011 study by the World Bank [10] projected air-pollution related health effects in UB using only outdoor air pollution as an indicator of exposure. The current analysis improves upon the methods used in that study by implementing more-advanced exposure estimation methods, a newer exposure-response technique, more-nuanced population and background disease projections, local household stove emissions data, detailed indoor air pollution measurement data, a baseline scenario inclusive of recent MCA efforts to distribute improved coal heating stoves, and an analysis inclusive of all UB districts. Our analysis also benefits from the emissions and survey data collected during the 2012–2013 MCA stove implementation project [9,22,23]. Nevertheless, the general trends identified in the World Bank report are in agreement with ours: heavy reductions in PM_2.5_ emissions, particularly those from household stoves, are needed to make appreciable impacts on the current pollution-related health burden in UB. In contrast, the high attributable disease rates remaining in our baseline, which included 100% replacement of traditional raw coal stoves with improved MCA stoves, and Pathway 1, which assumed even greater stove improvements and a switch by a large fraction of homes to CIU heating, suggests World Bank estimates that improved stoves can produce exposure reductions commensurate with a full transition to electric heating are unrealistic.

While few measurements exist with which to compare our values, a study [40] of indoor concentrations during the 2015 winter season in UB found geometric mean concentrations in apartments (52.8 μg/m^3^, 95% CI: 39–297 μg/m^3^) that were generally in agreement with our 2014 non-smoking clean apartment estimate (73 μg/m^3^). We expect our indoor estimates of homes heated with stoves to be more robust, as they were derived from the largest database of measured indoor concentrations of homes and gers in Ulaanbaatar, to date. Recently published measurements of indoor concentrations in gers using traditional stoves during the 2015 winter season (127.8 μg/m^3^, 95%CI: 86–190 μg/m^3^) are consistent with the value produced by our model for gers using traditional stoves (113.3 μg/m^3^) [40]. We are unaware of published measurements in cleanly heated houses for comparison.

There are several limitations to our study. Concerning the outdoor air quality modeling, Gaussian dispersion models are overly simplistic to capture all of the transport, dispersion, and terrain characteristics for UB wintertime conditions. Air quality modeling errors from the use of a Gaussian dispersion model are lumped together with emission inventory errors when calibrating the model to air quality observations. It is not clear how these errors propagate through to 2024. Un-modeled emission sources were assumed to have an impact of 10 μg/m^3^ and this simplification influences the exposure estimates, especially for Pathway 2 where modeled emission source contributions are low.

Indoor concentrations in ‘clean’ heating homes, which were calculated by applying infiltration factors to outdoor concentrations, may have propagated any error incurred by the outdoor model. These methods, which employed climate-relevant but non-local infiltration rates, could further be improved by future work characterizing local building infiltration rates. In addition, the linear model used to estimate wintertime indoor concentrations in stove heated homes, as described in S1 Text, proved a poor predictor of individual indoor concentrations with particular weakness predicting very high and low values (adj-r^2^ = 0.13). However, evidence suggests that stove type, SHS, and various structural characteristics associated with home type play important roles in shaping indoor concentration profiles in households using solid fuels [46–48]. In order to place measurement-based constraints on these relationships at the population level, the full model was kept, despite the low r^2^. More complex cross-validated modeling was attempted with these covariates using the SuperLearner machine learning package in R [49], but did not improve the fit. Moreover, the assumption that residential indoor PM_2.5_ concentrations reflect indoor concentrations in general is overly simplistic. Future research should elucidate PM_2.5_ concentrations throughout workplace, recreational, and other indoor environments in order to inform a more-nuanced population-wide indoor exposure model. This is especially important if a portion of the population works in high-exposure settings like coal mines.

The use of linear models to project background disease rates through 2024 may not reflect future trends in areas like rural to urban migration, economic development, regulatory shifts, and healthcare improvements which may have non-linear impacts on disease-influencing factors (e.g. introduction of pneumococcal conjugate vaccines). This general limitation is highlighted by the weak fit of the linear background disease model to historical data for several diseases, as demonstrated in S1 Text. Background disease projections may also have been substantially underestimated because of latent disease at the time of estimation. Because air pollution is a relatively new problem in UB (only gaining traction in the 1990s) and because of the rapid, recent influx of people from cleaner rural parts of Mongolia, the full impact of diseases that require several decades after exposure to develop, like lung cancer, were likely not fully represented in the background disease data used in our projections, biasing our burden estimates downward [35,50]. On the other hand, our decision to not consider exposure cessation lag in calculation of averted deaths and DALYs may have accelerated the accrual of estimated health benefits and resulted in modestly inflated estimates for the time periods given, except for ALRI which is an acute outcome. The effect of cessation lag on the final results may have been attenuated by the large fraction of burden caused by ALRI, especially DALYs.

Our BAU and alternative policy pathways were limited by gaps in the literature, too. For example, while recent government anti-smoking campaigns [30] suggest that smoking rates are likely to change, it is unclear how this will impact household SHS prevalence and related indoor concentrations. Implementation of a steady rate allowed the importance of addressing this issue to be clearly identified in the models. Information on the number of building structures and, thus, heating sources per home was also inconsistent. City census data seem to be reported in terms of primary residence type [17], while satellite imagery and field experience indicated that, in peri-urban areas, a considerable number of residences include multiple ger or house structures. This may have contributed to uncertainty in our exposure estimates which were, in part, based on the assumption that the indoor environment of each resident was dictated by a single combination of structure, heat source, and SHS presence.

Attributable burden estimates in UB may, in general, be underestimated. Evidence suggests that cold-air exposures may increase sensitivity to risk factors for cardiovascular diseases [51]. Ulaanbaatar’s temperatures are typically much colder than the regions that inform Burnett et al.’s exposure-response models [33], suggesting a risk misclassification biasing our burden estimates downward. Differential bias in the calculation of averted burden in our projections may have resulted from winter exposures dominating the annual average most in BAU, less in Pathway 1, and least in Pathway 2, thus leading to further underestimation of averted burden. It is also not clear at present whether pneumonia incidence is related to winter time exposures, or annual average exposures, although hospital records of pneumonia incidence indicate the majority of the burden is in winter months. The distinction becomes important for non-linear dose response curves where the wintertime exposures are on a much flatter section of the curve.

## Conclusion

Air pollution in Ulaanbaatar has reached a critical level, and immediate measures must be taken to reduce its health impacts on the city’s growing population. Current exposures are projected to produce unprecedented levels of respiratory illness, especially in children, and cardiovascular disease. Using some of the latest available exposure-response techniques and novel data on local emissions and indoor concentrations, this analysis is the most holistic view of population-wide air quality exposures in Ulaanbaatar to date. The results highlight the need for aggressive actions, including the elimination of residential coal burning and the reduction of current smoking rates, if the health burden of air pollution is to be reduced. Our conclusions support recent findings that PM_2.5_ emissions, especially from household heating, contribute substantially to mortality and morbidity from cardiovascular and pulmonary disease in the city. In addition, without efforts to moderate indoor concentrations, the full benefits of pollution reductions in UB will not be realized.

## Supporting information

S1 Text Supplemental text. S1 Text delivers more detail on the methods summarized in the primary publication. The details of the demographic, indoor air quality, and outdoor air quality models included in this analysis are beyond the scope and narrative of the primary publication, but are integral to the analysis and produce ancillary results novel in their own right. This supplement is intended to fill in gaps in the primary publication in order to provide a fully transparent account of the methods and sources used.
(DOCX)

S1 Fig Trends in family size (persons per household). Identified by the National Statistics Office of Mongolia for 2000–2010, and estimated using extrapolation and assumptions of the Total Fertility Rate for 2011–2030.
(TIF)

S2 Fig Projections of Ulaanbaatar household numbers stratified by area and home type. (TIF)

S3 Fig Adjusted disease-specific annual background mortality data (2006–2012) and projections (2014–2024). Projection models, model fits (α = 0.05), and model 95% Confidence Intervals are shown.
(EPS)

S4 Fig Wintertime (September through March) average emissions from residential heating stoves. (TIF)

S5 Fig Measured and modeled PM_2.5_ concentrations at four sites, January 22 –March 2, 2013. 10 μg/m^3^ was subtracted from each of the observed concentration values to adjust for sources not included in the modeling.
(TIF)

S1 Table Population projection estimates. S1 Table provides projection estimates for population (total and child) and household number (by household type) for 2013 through 2030 as applied in the manuscript.
(XLSX)

S2 Table City mortality data. S2 Table provides aggregated mortality data according to disease-specific ICD codes (see S1 Text) as analyzed in S1 Text and employed in the manuscript.
(XLSX)

S3 Table Population attributable burden. S3 Table provides mean, lower, and upper estimates of disease-specific population attributable (Risk) fraction, background disease burden, and attributable risk (deaths and Disability-Adjusted Life Years [DALYs]) by year and policy pathway (BAU, Pathway 1, Pathway 2) as analyzed in the main text and S1 Text.
(CSV)

S4 Table Sub-group exposures and risks. S4 Table provides exposure and disease-specific risk data for each population subgroup as analyzed in the main text and S1 Text.
(CSV)

S5 Table Outdoor model scaling. S5 Table provides PM_2.5_ mass concentration data for the period January 22—March 2, 2013 from the MCA-funded ambient fine particulate matter speciation study conducted by Ecography/Ecoworld.
(XLSX)

S6 Table Outdoor model output. S6 Table provides modeled PM_2.5_ mass concentration data for modeled districts of Ulaanbaatar, Mongolia by grid cell.
(XLSX)
