# Correction: Health assessment of future PM_2.5_ exposures from indoor, outdoor, and secondhand tobacco smoke concentrations under alternative policy pathways in Ulaanbaatar, Mongolia

**DOI:** 10.1371/journal.pone.0190432

**Published:** 2017-12-22

**Authors:** The PLOS ONE Staff

There are minor errors and missing information in the Acknowledgements section. The complete Acknowledgements are as follows: The authors are grateful to the National Statistics Office of Mongolia, the Statistics Department of Ulaanbaatar, and the Health Development Center of the Ministry of Health and Sports which provided access to various demographics and health databases. We are grateful to Social Impact and the Millennium Challenge Corporation for their open access databases of household measurements conducted as part of the impact evaluation of the Energy and Environment Projects. We thank Maria Hernandez, Ajay Pillarisetti, Paul Chung, and Alan Hubbard of the University of California, Berkeley and Nick Lam of the University of Illinois at Urbana-Champaign (formerly of the University of California, Berkeley) for their advice and generous assistance during the project. We acknowledge Boldkhuu Nanzad of the Ministry of Energy of Mongolia for advocating for our research, and appreciate Berkeley Air Monitoring Group for facilitating financial arrangements. We also acknowledge that the final analysis benefits from comments made by many participants at a workshop presenting preliminary results conducted as part of the impact evaluation of the Energy and Environment Projects. This material is based upon work supported by the National Science Foundation Integrative Graduate Education and Research Traineeship under Grant No. DGE-1144885. Any opinions, findings, and conclusions or recommendations expressed in this material are those of the author(s) and do not necessarily reflect the views of the National Science Foundation. LDH was supported by this NSF traineeship during part of the write up and analysis phases of this project. This NSF traineeship support was generously provided through the Berkeley Center for Green Chemistry. During the peer-review process, a modified version of this paper was included as a chapter in a doctoral dissertation at the University of California, Berkeley. The full dissertation, which includes five total chapters and is titled “A breath of fresher air: improving methods for PM_2.5_ exposure assessment from Mongolia to California” by Lawson Andrew Hill, will eventually be available at ProQuest/UMI or the University of California’s online repository, “eScholarship.”

There is an error in the Methods section in the subsection called Estimating health effects. The correct term for “chronic obstructive pulmonary disorder” is “chronic obstructive pulmonary disease.”

There is an error in the third sentence of the Abstract. The word “assesses” should read “assess.”
