# Supplementary material for: Health assessment of future PM2.5 exposures from indoor, outdoor, and secondhand tobacco smoke concentrations under alternative policy pathways in Ulaanbaatar, Mongolia
Source: PLoS One. 2017 Oct 31;12(10):e0186834. doi: 10.1371/journal.pone.0186834 (PMC5663421; doi:10.1371/journal.pone.0186834)
Supplement: S1 Text — delivers more detail on the methods summarized in the primary publication. The details of the demographic, indoor air quality, and outdoor air quality models included in this analysis are beyond the scope and narrative of the primary publication, but are integral to the analysis and produce ancillary results novel in their own right. This supplement is intended to fill in gaps in the primary publication in order to provide a fully transparent account of the methods and sources used. (DOCX) [file pone.0186834.s001.docx]

# S1 Text. Supplemental text.

## Demographic conditions

### Projecting population and household numbers

Citywide estimates of population and household size were used to calculate household numbers and disease burdens. Population projections (total population and population < 5 years old) for Ulaanbaatar (UB) for 2010, 2015, 2020, 2025, and 2030 were taken as the “medium growth” (version 1b) projections identified in the 2010 Population and Housing Census of Mongolia Report [1]. Annual population estimates for relevant interim years (2014-2024) were estimated by linearly interpolating the 5-year projections in Microsoft Excel for Mac 2011 and 2016. Detailed information on the spatial distribution of population and household number by household type was obtained from 2012 city census data [2].

Projections were made for the number of homes by type throughout Ulaanbaatar. Household types most relevant to the Ulaanbaatar context were identified as gers, single family houses, and multi-family apartments, as described in the main text. Projections for the number of total households in each year were unavailable, and so were estimated from family size and total population, assuming an average of one family per household. Family size was extrapolated by fitting a curve to historical trends (2000-2010) obtained from Mongolia’s Annual Statistical Yearbook series (S1 Fig.) [3–5]. Because of Ulaanbaatar’s recent rapid rate of decrease in family size, a linear extrapolation resulted in unrealistically low family sizes in future years. Thus, total fertility rates (TFR) were used to infer a reasonable lower limit.

TFR is defined by the United Nations (UN) as the average number of children a hypothetical cohort of women would have at the end of their reproductive period if they were subject during their whole lives to the fertility rates of a given period and if they were not subject to mortality. While modeling the UN’s 5-year TFR estimates for Mongolia against the 10 available years of household size data yielded no discernable relationship, we assumed that trends in family size would follow the national TFR. Mongolia’s TFR has experienced a dramatic decrease in recent decades, but is expected to level off. The UN suggests that Mongolia’s TFR will become stable at near-Western levels by 2030 [6]. This suggests that while the estimated rate of decline in household size observed between 2000 and 2010 was steep and linear, it is likely to level off in the near future. For this reason, a trigonometric curve was fit to past data that would approximate a near-term asymptotic approach of 2.6 persons/home, the average 2010 US household size [7]. Eureqa Formulize [8] was used to fit this curve. Household sizes for individual years during 2010-2030 were then taken from this curve with the assumption of one family per household (S1 Fig.). Citywide household number was estimated by dividing the expected size of Ulaanbaatar’s population in each year by the average household size.

Gers and single-family houses are typically located in regions identified by the Statistics Department of Ulaanbaatar as “ger areas,” while apartment households are typically in “apartment areas.” Projections of the proportion of Ulaanbaatar residents living in the ger areas were provided by the Ulaanbaatar 2020 Master Plan and Development Approaches for 2030 report [9]. Annual trends were linearly interpolated in Microsoft Excel for Mac 2011 and 2016. The proportion of all households located in ger areas was taken directly from the population interpolations. All other households were assumed in the apartment areas. Approximately 99% of households in the apartment areas in 2012 were multi-unit apartments [2]. The remaining 1% of households was classified as “luxury house” or “homeless”; their household heating emissions and exposures were not explicitly calculated, and were thus assigned the population-weighted averages of all other households. Statistics Department figures show that in the ger areas, families were about evenly split between gers and houses. In 2012, families living in gers accounted for 44.4% of ger area households, while families living in houses accounted for 54.4% of ger area households [2]. The remaining ger area household types (~ 1%) were assigned the population-weighted average heating emissions and exposures of all other household types. Projections for the proportion of ger area households as gers vs. houses were unavailable, and so the 2012 proportion was assumed constant over time. The final projections of household number by type and location are shown in S2 Fig.

### Projecting background disease rates

During analysis, a considerable discrepancy was discovered between locally identified death rates and nationally identified death rates. Recent estimates of Mongolia-wide disease-specific mortality rates [10] show values that are between 50% and 400% higher than those identified in the data for Ulaanbaatar received from the Ministry of Health and Sports. While national disease rates should not exactly mirror those of the capital city, they are expected to be similar. It is suspected these discrepancies arise from the inclusion of “garbage codes”, or improperly coded deaths, in the raw Ministry of Health and Sports dataset. Garbage codes are a well-known phenomenon and occur frequently all over the world [11]. International teams like the Institute for Health Metrics and Evaluation (IHME) employ rigorous statistical and diagnostic methods to redistribute such deaths to their probable underlying causes. This results in disease-specific mortality estimates that more accurately represent true rates. Disease-specific data employed in this analysis were thus adjusted using IHME values [10], as discussed in greater detail below.

Mortality data for the capital city were modeled through 2024 using historical data for 2006-2012 provided by the Health Development Center of the Ministry of Health and Sports in conjunction with the Mongolian National University of Medical Sciences. Deaths for 2006-2012 matching the ICD-10 codes used to calculate PM_2.5_-related illness in the IHME 2010 Global Burden of Disease Study were obtained [11]— Ischemic Heart Disease was defined as mortality from ICD-10 codes I20-I25; Stroke, from I63, I65 - I67 (excluding I67.4), I69.3, I60-I62, I69.0 - I69.2, and I67.4; Lung Cancer, from C33- C34, D02.1-D02.2, and D38.1; Chronic Obstructive Pulmonary Disease, from J40 – J44, and J47; and Acute Lower Respiratory Infections in children < 5 years old, from J09 – J11 , J13, J14, J12.1, J12 (excluding J12.1), J15 – J22, J85, and P23. Linear models were chosen to produce consistent, parsimonious projections of total mortality in UB from each disease in each year of the study. Disease-specific mortality models were created in R [12] and took the form of Equation S1, where β_0,k_ is the y-intercept for disease “k” and β_1,k_ is the regression coefficient for the effect of year ”j" on the number of deaths from disease “k”. The model was adjusted for the discrepancy between local and national mortality data using “φ”, the ratio of IHME-reported national death rate to UB-specific death rate (Table A). The results of the model are shown in Table B. Ratios (φ) were created for 2010, which was the only year for which both IHME and Ministry of Health and Sports estimates were available. Values for the 2010 UB total population and child (0-4 year) population were taken as the “medium growth” estimates (version 1b) from the 2010 Population and Housing Census of Mongolia Report [1] for consistency with previously discussed demographic estimation methods. Adjusted estimates of background disease values in each year are provided in Table C, and adjusted disease-specific mortality models are shown in S3 Fig. This figure shows that not all mortality models had a significant fit, but the resulting estimates were reasonable given the low sample sizes. Population was explored as a covariate, but overall did not produce a better set of models.

${Adjusted Total Mortality}_{j,k}=\left( \beta_{0,k} + \beta_{1,k}\cdot\left( Year j \right) \right)\cdot\phi_{k}$ (S1)

**Table A. Rate adjustment factor (φ) and background mortality rates for 2010 as tabulated for UB from Ministry of Health and Sports data for UB and as taken from IHME [10] national rates**

| Disease | UB 2010 Deaths per 100,000 | Mongolia 2010 Deaths per 100,000 | Ratio of National Rate to UB rate (φ) |
| --- | --- | --- | --- |
| Lung Cancer | 12.12 | 18.14 | 1.50 |
| ALRI | 78.22 | 276.82 | 3.54 |
| COPD | 4.45 | 21.66 | 4.87 |
| Ischemic Heart Disease | 86.13 | 164.19 | 1.91 |
| Stroke | 76.89 | 124.22 | 1.62 |

**Table B. Unadjusted background disease rate projection models, by disease**

|  | β0 | SE | β1 | SE | p-value | *φ* |
| --- | --- | --- | --- | --- | --- | --- |
| **Lung Cancer** | -7767 | 7267 | 3.93 | 3.62 | 0.33 | *1.50* |
| **ALRI** | -10541 | 7341 | 5.29 | 3.65 | 0.21 | *3.54* |
| **COPD** | -11509 | 3451 | 5.75 | 1.72 | 0.02 | *4.87* |
| **Ischemic Heart Disease** | -89524 | 35962 | 45.0 | 17.9 | 0.05 | *1.91* |
| **Stroke** | -22871 | 20113 | 11.80 | 10.01 | 0.29 | *1.62* |

**Table C. Adjusted estimates of background disease in Ulaanbaatar, 2014-2024**

|  | Lung Cancer | | ALRI (0-4 years) | | COPD | | | Ischemic Heart Disease | | | Stroke | |  |
| --- | --- | --- | --- | --- | --- | --- | --- | --- | --- | --- | --- | --- | --- |
| Year | *Deaths* | *DALYs* | *Deaths* | *DALYs* | *Deaths* | *DALYs* | *Deaths* | | *DALYs* | *Deaths* | | *DALYs* | |
| 2014 | 218 | 5,463 | 371 | 31,760 | 350 | 13,700 | 1,974 | | 43,977 | 1,402 | | 32,220 | |
| 2015 | 224 | 5,610 | 389 | 33,364 | 378 | 14,796 | 2,060 | | 45,890 | 1,421 | | 32,659 | |
| 2016 | 230 | 5,758 | 408 | 34,967 | 406 | 15,892 | 2,146 | | 47,803 | 1,440 | | 33,097 | |
| 2017 | 236 | 5,905 | 427 | 36,570 | 434 | 16,987 | 2,232 | | 49,716 | 1,459 | | 33,536 | |
| 2018 | 242 | 6,052 | 446 | 38,173 | 462 | 18,083 | 2,318 | | 51,628 | 1,478 | | 33,975 | |
| 2019 | 248 | 6,200 | 464 | 39,776 | 490 | 19,179 | 2,404 | | 53,541 | 1,497 | | 34,414 | |
| 2020 | 254 | 6,347 | 483 | 41,379 | 518 | 20,275 | 2,490 | | 55,454 | 1,516 | | 34,853 | |
| 2021 | 260 | 6,494 | 502 | 42,983 | 546 | 21,370 | 2,575 | | 57,367 | 1,536 | | 35,291 | |
| 2022 | 266 | 6,642 | 520 | 44,586 | 574 | 22,466 | 2,661 | | 59,280 | 1,555 | | 35,730 | |
| 2023 | 272 | 6,789 | 539 | 46,189 | 602 | 23,562 | 2,747 | | 61,193 | 1,574 | | 36,169 | |
| 2024 | 277 | 6,936 | 558 | 47,792 | 630 | 24,658 | 2,833 | | 63,106 | 1,593 | | 36,608 | |

## Indoor air quality estimates

### Indoor air quality for MCA stove users

Linear models were used to estimate indoor concentrations in stove-heated homes from data collected as part of the impact evaluation of the Millennium Challenge Account (MCA) Mongolia Energy and Environment Project Energy Efficient Stove Subsidy Program conducted by Social Impact (SI) for the Millennium Challenge Corporation (MCC) [13–15]. Details of the larger study can be found in that report [13]. Briefly, overnight indoor PM_2.5_ concentrations were measured using filter-based techniques in gers and houses throughout UB during the winter of 2012-2013. Measurements were spread over three distinct winter phases representing early, mid, and late winter. The linear model was created in R [12] and took the form of Equation S2, where Indoor PM_2.5_ is the overnight average indoor concentration of PM_2.5_ (mg/m^3^), β_0_ is the intercept representing a non-smoking house with an MCA project stove during the first measurement phase of the emissions study, β_1_ is the impact of using a traditional stove rather than an MCA stove, β_2_ is the impact of the presence of a tobacco smoker in the home, β_3_ is the impact of a ger environment rather than a house, and β_4 and_ β_5_ are the additional impacts of the measurement being taken during the second and third measurement phases, respectively.

$log(Indoor {PM}_{2.5}) = \beta_{0}+ \beta_{1}*Traditional+ \beta_{2}*ETS+ \beta_{3}*Ger+ \beta_{4}*Phase2+ \beta_{5}*Phase3$ (S2)

These variables were chosen as evidence suggests they should have a considerable impact on indoor concentrations at the population level [16–18]. A log-transformation was made to indoor concentration, as required by the log-normal distribution of the data (Shapiro-Wilk p < 0.0001). Four data points from the original MCC dataset were excluded because of inconsistency between various household type indicators, and sixteen observations were excluded because at least one of the variables of interest was missing, leaving a total model sample size of 196 for the modeling. The results of Equation S2 are detailed in Table D. Average indoor wintertime concentrations by home, stove, and secondhand tobacco smoke (SHS) as applied in the exposure model discussed in the main text were calculated by averaging model results across the three wintertime study phases.

**Table D. Household indoor log-PM_2.5_ concentration model**

|  | **Estimate** | **Std. Error** | | **p-value** |  |
| --- | --- | --- | --- | --- | --- |
| **β_0_** | -2.21 | 0.16 | | < 0.0001 |  |
| **β_1_ (Traditional Stove)** | 0.057 | 0.090 | | 0.53 |  |
| **β_2_ (SHS)** | 0.16 | 0.082 | | 0.059 |  |
| **β_3_ (Ger Dwelling)** | -0.10 | 0.082 | | 0.22 |  |
| **β_4_ (Study Phase 2)** | 0.33 | 0.15 | | 0.031 |  |
| **β_5_ (Study Phase 3)** | -0.15 | 0.16 | | 0.34 |  |
| Model Adjusted R^2^: 0.132 | | | Model p-value: < 0.0001 | | |

### Indoor air quality in homes using low pressure boilers, heat only boilers, and other stoves

Low pressure boilers and semi-coke coal stoves are widely heralded by the public for their improvements in efficiency and functionality over traditional coal stoves, but little data on their contributions to indoor PM_2.5_ exist. It is unreasonable to suggest completely clean function. For the sake of simplicity and a lack of data, we assigned the same indoor PM_2.5_ concentrations to homes with low pressure boilers and semi-coke coal stoves as were assigned to those with MCA stoves, by home type. As discussed elsewhere, Future Tech stoves were assumed to produce a 20% reduction in indoor PM_2.5_ concentrations over MCA stoves, by home type. For BAU and both alternative policy pathways, the contribution of SHS to indoor concentrations in gers was assumed the same as the contribution modeled from SHS in gers using MCA stoves (18.1 µg/m^3^). The contribution of SHS to indoor concentrations in apartments and houses was assumed the same as that modeled in MCA stove houses (20.0 µg/m^3^), as our model did not provide data on SHS in apartments and because apartments are structurally more similar to houses than gers.

### Infiltration efficiency

Data on the infiltration efficiencies of houses and apartments in the context of Ulaanbaatar were lacking, so local infiltration efficiencies were estimated from geographically similar regions. Seasonal average infiltration efficiencies for PM_2.5_ in apartment buildings and houses were taken from EXPOLIS, a study that included the calculation of infiltration efficiency in several major cities [19,20]. We used the average seasonal infiltration efficiencies specifically reported for buildings in urban Helsinki, because it is the EXPOLIS city with a climate most similar to that of UB. From these data, the average of the infiltration efficiencies reported for the spring (March-May) and summer (June-August) seasons (64%) was used in our summertime (April - September) calculations, and the average of the infiltration efficiencies reported for the autumn (September - November) and winter (December - February) seasons (53%) was used in our wintertime (October-March) calculations. These values are consistent with infiltration efficiencies found in similar home types, climates and seasons [21].

Unlike houses and apartments, the ventilation characteristics of which are more limited, gers have ceiling flaps and large doorways that are left open for much of the summer months. This is likely to result in high ventilation rates and a virtual elimination of filtration related to airflow through building casings. Thus, a summer infiltration efficiency of 100% was applied to gers. A wintertime infiltration efficiency of 70% was estimated from blower door tests performed as part of the United Nations Development Programme – Global Environment Facility Commercialization of Super-Insulated Buildings in Mongolia project (MON/99/G35), the results of which were communicated to us by a collaborator on the project, Munkhbayar B. at the Mongolian University of Science and Technology. The tests provided the number of winter time air changes per hour at 50 Pascals of pressure difference (n_50_) for gers with modest insulation and fly cover: 45 air changes per hour. The n_50_ air change rate was converted to a natural air change rate of 2.25 air changes per hour using the methods described in [22]. This natural air change rate was translated into an estimate of wintertime PM_2.5_ infiltration efficiency of 70% using a curve presented in [23] for the translation of general air exchange rates into PM_2.5_ infiltration rates.

## Residential heating stoves emissions field

S4 Fig. shows the 2014 Base scenario wintertime (September through March) average emission rate from coal-fired residential heating stoves as projected onto 1 km × 1 km grids.

## Scaling outdoor ambient PM_2.5_ concentration models

Ambient air quality model performance was evaluated using model-to-monitor comparisons. Limited outdoor PM_2.5_ data were available for this comparison. For example, during the 2012-2013 heating season PM_2.5_ mass concentration data were collected with high data completeness by the National Agency for Meteorology, Hydrology, and Environment Monitoring at one location – air quality monitoring station #2 (UB02). This site was next to a major roadway and likely suffered high impacts from local traffic that could not be resolved by the model. Thus, outdoor PM_2.5_ data collected by Ecography and Ecoworld under contract from MCA-Mongolia were used for the comparison. The sampling locations, methodology [14,15], and key results are detailed in the full SI project report [13]. Their data from January 22 to March 2, 2013 were used for the model-to-monitor comparison with 19 samples per site. The 2014 projected inventory was used except that residential stove emissions were calculated under the assumption of full-penetration of MCA stoves as defined in the main text.

The measured average PM_2.5_ concentrations at each site, shown by the single-crossed bars in S5 Fig., demonstrate high spatial variability with up to a 50% difference between sites. The sample time period from the full MCA project was also modeled; average concentrations including all days between January 22 and March 2 are shown by the solid black bars in S5 Fig. [13]. Modeled concentrations were much lower than the measured values and were less variable between sites. There were several possible reasons for these differences including, but not limited to, the emissions for these sources being underestimated and the model not being able to account for the trapping and accumulation of emissions from one hour to the next. The model was reconciled to the measurement data by increasing the residential stove, HOB, and motor vehicle emissions by a factor of 2.85, which was the value of the four measured-to-modeled concentration ratios. The cross-hatched bars in S5 Fig. show the modeled PM_2.5_ concentrations after this scaling. Assuming the only error was in the emissions inventory, the nearly threefold increase of the projected JICA 2010 inventory was still lower than the inventory projected by Guttikunda et al. for 2010 for each of these source categories [24,25]. While the scaling increases the emission inventory for these sources by about a factor of three, this places the effective emissions between those projected from the year 2010 inventories prepared by JICA and Guttikunda et al. Thus, the scaled emissions were deemed reasonable because they were bounded by the best available inventories. Power plant emissions were not scaled because the JICA and Guttikunda et al. inventories are relatively similar and emissions from tall stacks are less likely to be trapped and accumulate at ground level. This residential stove, HOB, and motor vehicle emissions scaling was applied during modeling to BAU and the two alternative pathways.

## References

1. National Statistics Office of Mongolia. The 2010 population and housing census of Mongolia. Ulaanbaatar, Mongolia: National Statistics Office of Mongolia; 2012.

2. Statistics Department of Ulaanbaatar. Population and household census 2012. Ulaanbaatar: Statistics Department of Ulaanbaatar; 2013.

3. National Statistics Office of Mongolia. Mongolian statistical yearbook 2002. Ulaanbaatar: National Statistics Office of Mongolia; 2003.

4. National Statistics Office of Mongolia. Mongolian statistical yearbook 2006. Ulaanbaatar: National Statistics Office of Mongolia; 2007.

5. National Statistics Office of Mongolia. Mongolian statistical yearbook 2010. Ulaanbaatar: National Statistics Office of Mongolia; 2011.

6. United Nations Department of Economic and Social Affairs: Population Division. World population prospects the 2012 revision volume I :comprehensive tables. New York, NY: United Nations; 2013.

7. United States Bureau of the Census. Households and families: 2010. Census 2010 brief C2010BR-14. 2012. doi:C2010BR-14

8. Schmidt M, Lipson H. Eureqa (Version 0.99.6 beta). 2013.

9. Tcakhiur S, Narangerel G, Ganbat P. Ulaanbaatar 2020 master plan and development approaches for 2030. Ulaanbaatar: Ministry of Construction and Urban Development and the Ulaanbaatar City Mayor’s Office; 2013.

10. Lim SS, Vos T, Flaxman AD, Danaei G, Shibuya K, Adair-Rohani H, et al. A comparative risk assessment of burden of disease and injury attributable to 67 risk factors and risk factor clusters in 21 regions, 1990-2010: a systematic analysis for the Global Burden of Disease Study 2010. Lancet. 2012;380: 2224–60. doi:10.1016/S0140-6736(12)61766-8

11. Lozano R, Naghavi M, Foreman K, Lim S, Shibuya K, Aboyans V, et al. Global and regional mortality from 235 causes of death for 20 age groups in 1990 and 2010: a systematic analysis for the Global Burden of Disease Study 2010. Lancet. 2012;380: 2095–128. doi:10.1016/S0140-6736(12)61728-0

12. R Core Team. R: A language and environment for statistical computing. Vienna: R Foundation for Statistical Computing; 2016.

13. Greene L, Turner J, Edwards R, Cutler N, Duthie M, Rostapshova O. Impact evaluation results of the MCA Mongolia Energy and Environment Project Energy-Efficient Stove Subsidy Program. Arlington; 2014.

14. Greene L, Turner J, Edwards R, Cutler N, Duthie M, Rostapshova O. Mongolia - energy and environment project, stove subsidies component: get microdata. In: Impact evaluation results of the MCA Mongolia energy and environment project energy-efficient stove subsidy program [Internet]. 2014 [cited 9 Aug 2016]. Available from: https://data.mcc.gov/evaluations/index.php/catalog/133/get_microdata

15. Greene L, Turner J, Edwards R, Cutler N, Duthie M, Rostapshova O. Social Impact 2012-2013 household survey data in support of the impact evaluation of the MCA Mongolia Energy and Environment Project Energy-Efficient Stove Subsidy Program. Arlington: Social Impact; 2014.

16. Balakrishnan K, Ghosh S, Ganguli B, Sambandam S, Bruce N, Barnes DF, et al. State and national household concentrations of PM2.5 from solid cookfuel use: results from measurements and modeling in India for estimation of the global burden of disease. Environ Heal. Environmental Health; 2013;12: 77. doi:10.1186/1476-069X-12-77

17. Li T, Cao S, Fan D, Zhang Y, Wang B, Zhao X, et al. Household concentrations and personal exposure of PM2.5 among urban residents using different cooking fuels. Sci Total Environ. Elsevier B.V.; 2016;548–549: 6–12. doi:10.1016/j.scitotenv.2016.01.038

18. Chowdhury Z, Campanella L, Gray C, Al Masud A, Marter-Kenyon J, Pennise D, et al. Measurement and modeling of indoor air pollution in rural households with multiple stove interventions in Yunnan, China. Atmos Environ. Elsevier Ltd; 2013;67: 161–169. doi:10.1016/j.atmosenv.2012.10.041

19. Hänninen OO, Lebret E, Ilacqua V, Katsouyanni K, Künzli N, Srám RJ, et al. Infiltration of ambient PM2.5 and levels of indoor generated non-ETS PM2.5 in residences of four European cities. Atmos Environ. 2004;38: 6411–6423. doi:10.1016/j.atmosenv.2004.07.015

20. Jantunen MJ, Katsouyanni K, Lebret E, Maroni M, Saarela K, Zmirou D. Final report: air pollution exposures in European cities: the EXPOLIS study. Prague; 1998.

21. Long CM, Suh HH, Catalano PJ, Koutrakis P. Using time- and size-resolved particulate data to quantify indoor penetration and deposition behavior. Environ Sci Technol. 2001;35: 2089–2099. doi:10.1021/es001477d

22. Sherman MH. Estimation of infiltration from leakage and climate indicators. Energy Build. 1987;10: 81–86. doi:10.1016/0378-7788(87)90008-9

23. Williams R, Suggs J, Rea A, Sheldon L, Rodes C, Thornburg J. The Research Triangle Park particulate matter panel study: modeling ambient source contribution to personal and residential PM mass concentrations. Atmos Environ. 2003;37: 5365–5378. doi:10.1016/j.atmosenv.2003.09.010

24. Japan International Cooperation Agency. Capacity development project for air pollution control in Ulaanbaatar city Mongolia, final report. Tokyo: Japan International Cooperation Agency; 2013.

25. Guttikunda SK, Lodoysamba S, Bulgansaikhan B, Dashdondog B. Particulate pollution in Ulaanbaatar, Mongolia. Air Qual Atmos Heal. 2013;6: 589–601. doi:10.1007/s11869-013-0198-7
