# Supplementary figures and images for: Health assessment of future PM2.5 exposures from indoor, outdoor, and secondhand tobacco smoke concentrations under alternative policy pathways in Ulaanbaatar, Mongolia
Source: PLoS One. 2017 Oct 31;12(10):e0186834. doi: 10.1371/journal.pone.0186834 (PMC5663421; doi:10.1371/journal.pone.0186834)

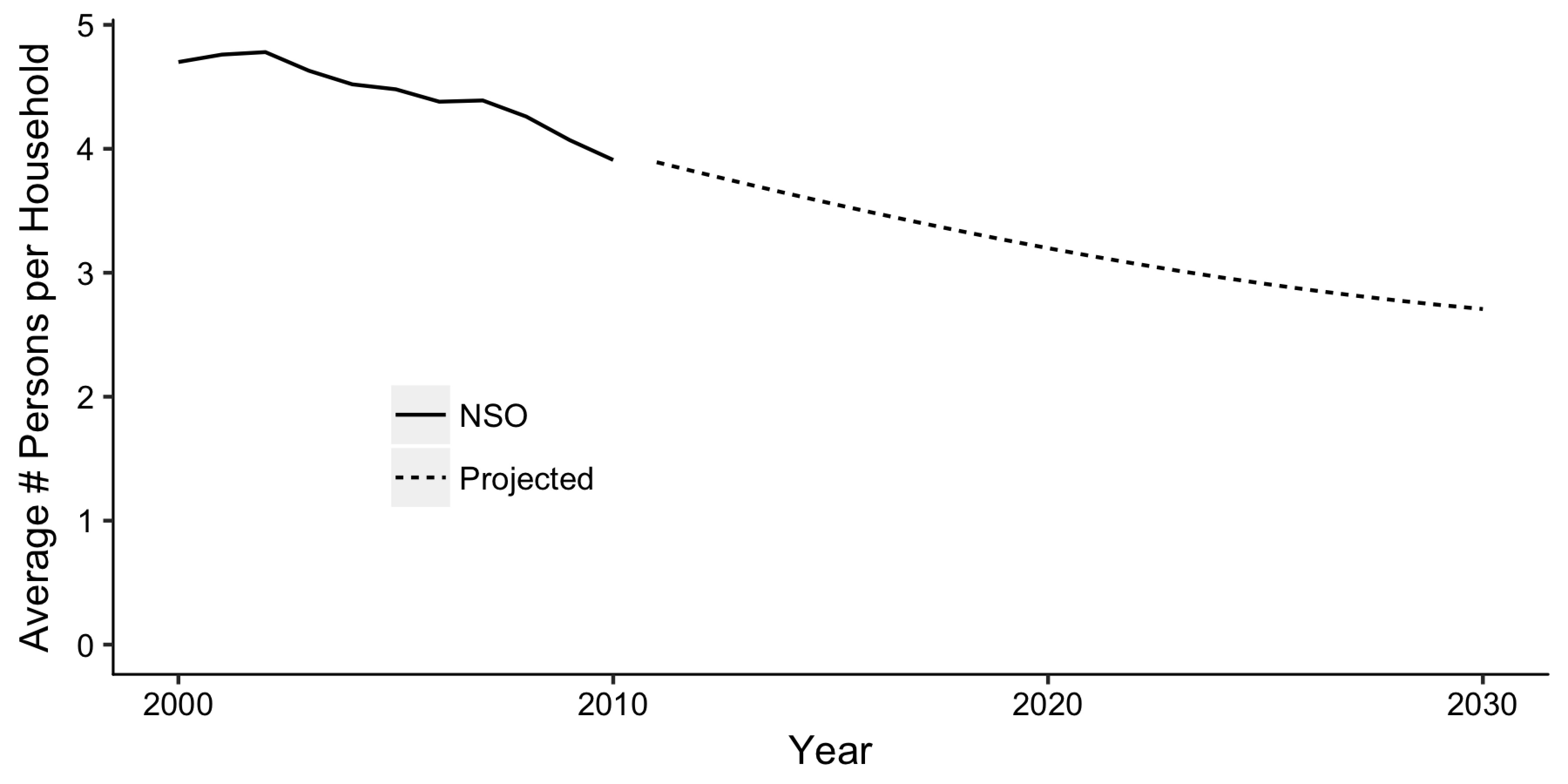

Supplement: S1 Fig — Identified by the National Statistics Office of Mongolia for 2000–2010, and estimated using extrapolation and assumptions of the Total Fertility Rate for 2011–2030. (TIF) [file pone.0186834.s002.tif]

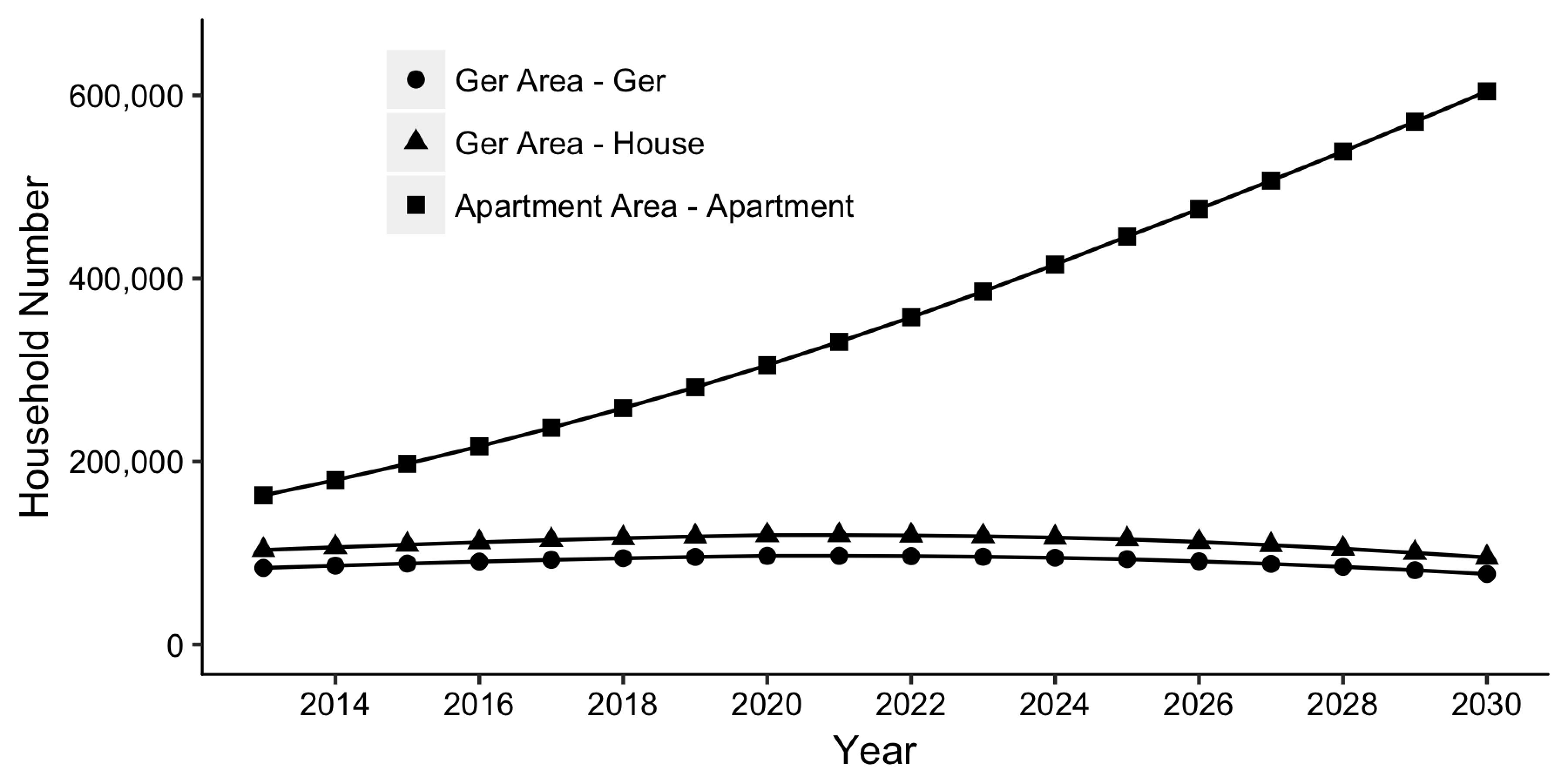

Supplement: S2 Fig — (TIF) [file pone.0186834.s003.tif]

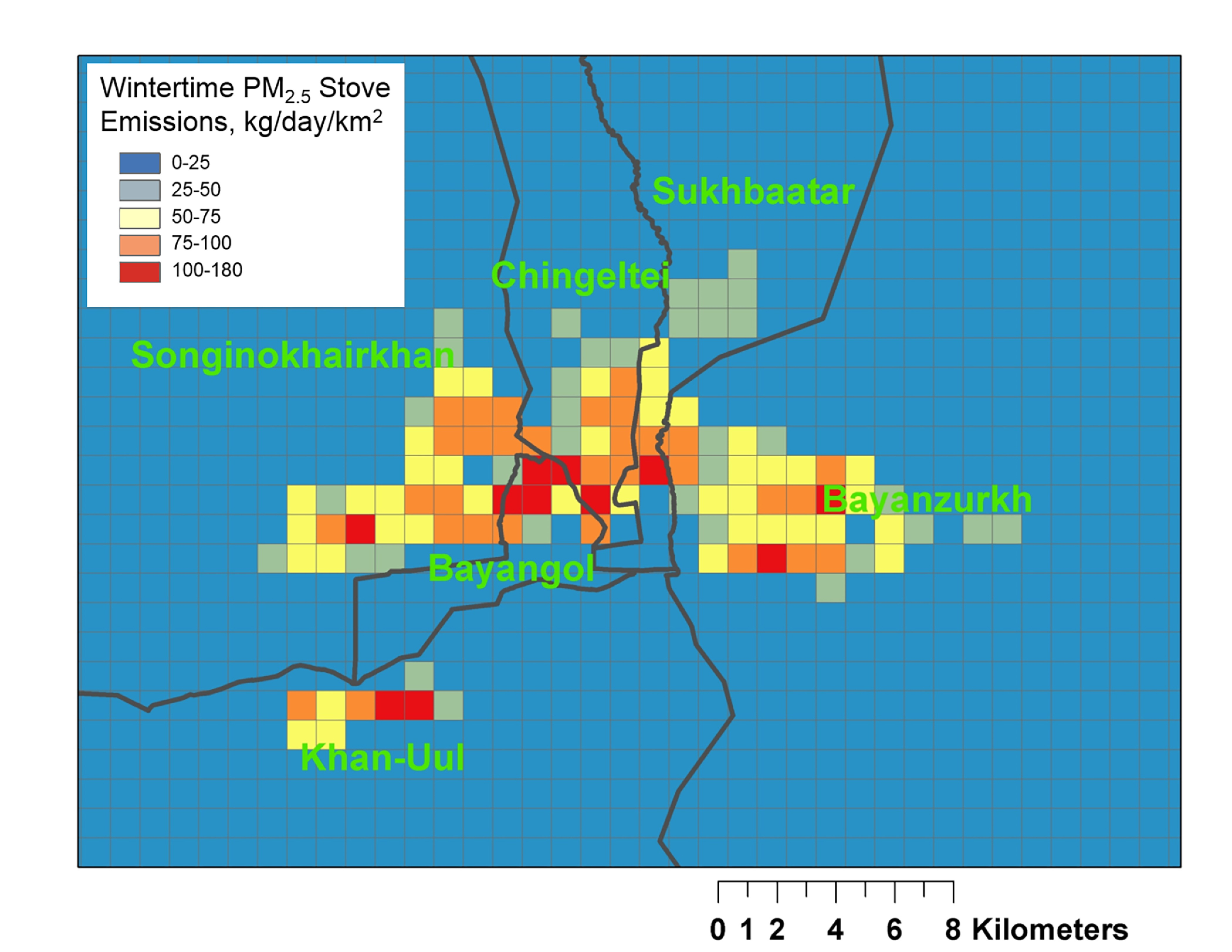

Supplement: S4 Fig — (TIF) [file pone.0186834.s005.tif]

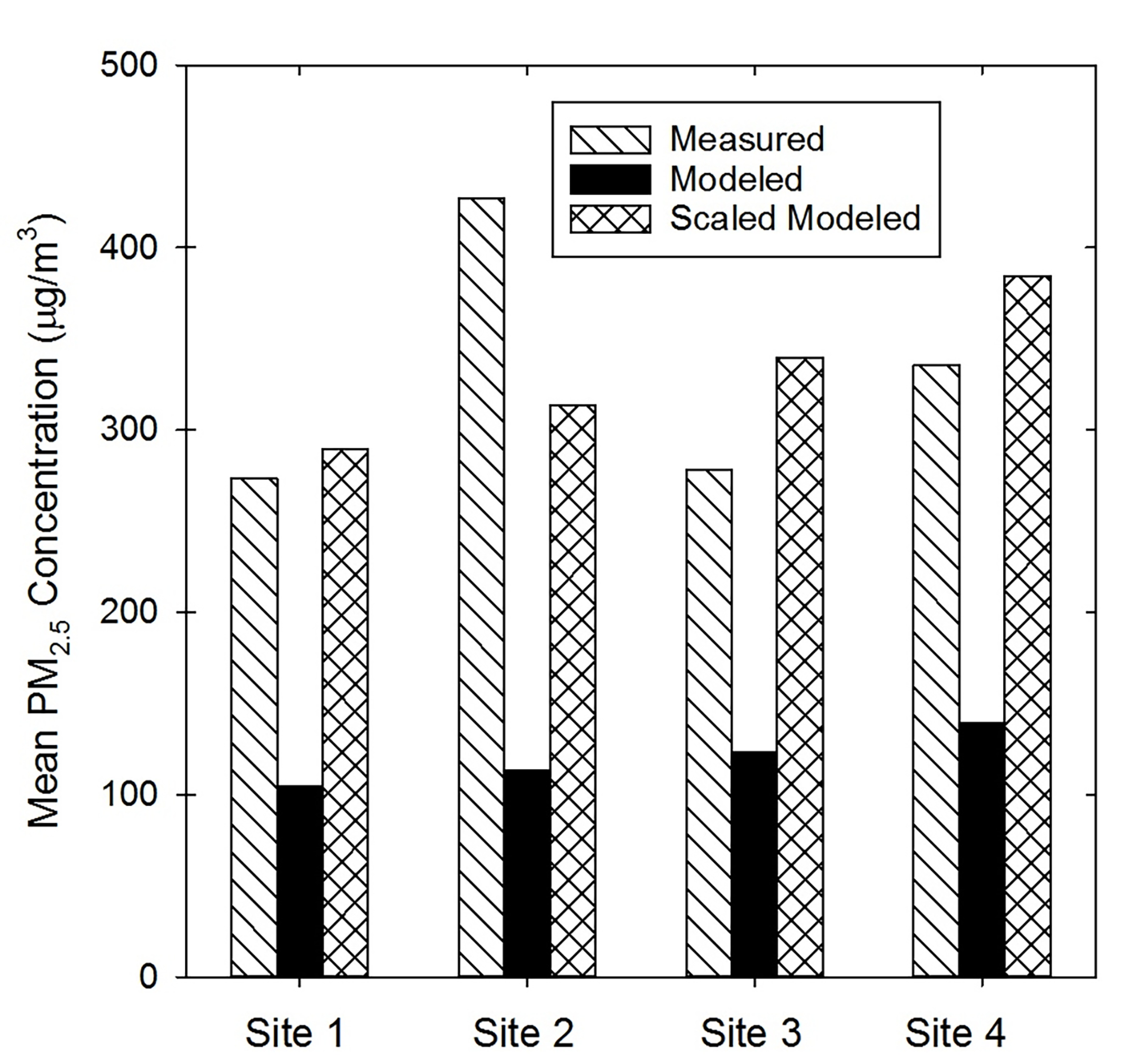

Supplement: S5 Fig — 10 μg/m3 was subtracted from each of the observed concentration values to adjust for sources not included in the modeling. (TIF) [file pone.0186834.s006.tif]
